# The *Hox* genes *Ultrabithorax* and *abdominal-A* specify three different types of abdominal appendage in the springtail *Orchesella cincta* (Collembola)

**DOI:** 10.1186/2041-9139-5-2

**Published:** 2014-01-07

**Authors:** Barbora Konopova, Michael Akam

**Affiliations:** Laboratory for Development and Evolution, Department of Zoology, University of Cambridge, Downing Street, Cambridge, CB2 3EJ UK

**Keywords:** *abdominal-A*, Appendage specification, Collembola, Evolution, *Hox*, Homeosis, RNA interference, Segment identity, *Ultrabithorax*

## Abstract

**Background:**

In *Drosophila* and many other insects, the *Hox* genes *Ultrabithorax (Ubx)* and *abdominal-A (abd-A)* suppress limb formation on most or all segments of the abdomen. However, a number of basal hexapod lineages retain multiple appendages on the abdomen. In the collembolans or springtails, three abdominal segments develop specialized organs that originate from paired appendage primordia which fuse at the midline: the first abdominal segment bears the collophore (ventral tube), involved in osmoregulation; the fourth segment bears the furca, the leaping organ, and the third segment bears the retinaculum, which retains the furca at rest. *Ubx* and *abd-A* are known to be expressed in the springtail abdomen, but what role they play in specifying these distinct abdominal appendages is not known. This is largely because no genetic model has been established in collembolans or any other non-insect hexapod.

**Results:**

We have developed a convenient method for laboratory culture of the collembolan *Orchesella cincta* on defined media, a method for *in-situ* hybridization to embryos and a procedure for gene knockdown by parental injection of double-stranded RNA (RNAi). We show that *Orchesella Ubx* transcripts are detectable in the first to third abdominal segments, and *abd-A* transcripts in the second to fourth segments. Knockdown of *Oc-Ubx* leads to the homeotic transformation of the collophore into a pair of walking legs (a more anterior identity) but the retinaculum into a furca (a more posterior identity). Knockdown of *Oc-abd-A* leads to the transformation of the retinaculum into a collophore and of the furca into legs (both anterior transformations). Simultaneous silencing of both *Oc-Ubx* and *Oc-abd-A* transformed all three of these appendages into paired legs, but did not cause appendages to develop on the second, or on the most posterior abdominal segments.

**Conclusions:**

We conclude that, in *Orchesella*, *Oc-Ubx* alone specifies the collophore on the first and *Oc-abd-A* alone specifies the furca on the fourth abdominal segment. *Oc-Ubx* and *Oc-abd-A* function together, apparently combinatorially, to specify the retinaculum on the third segment. The efficiency of RNAi in *Orchesella* makes this an attractive model for further genetic studies of development and physiology in basal hexapods.

**Electronic supplementary material:**

The online version of this article (doi:10.1186/2041-9139-5-2) contains supplementary material, which is available to authorized users.

## Background

Springtails (Collembola), Protura and Diplura, together with the insects proper, form a clade of arthropods called the Hexapoda. A defining feature of hexapods is that their body has six legs, a pair growing from each of the three thoracic segments [1, 2]. In larvae and adults of the more basal hexapod lineages, such as the springtails, Protura, Diplura and wingless insects, small limbs or other appendages also develop on the abdomen. In this respect, these basal lineages resemble many crustaceans, from some lineage of which the Hexapoda originated [2–4]. In the winged insects (Pterygota), abdominal appendages may develop in larvae, but are lost during metamorphosis to the adult.

**Figure 1:**
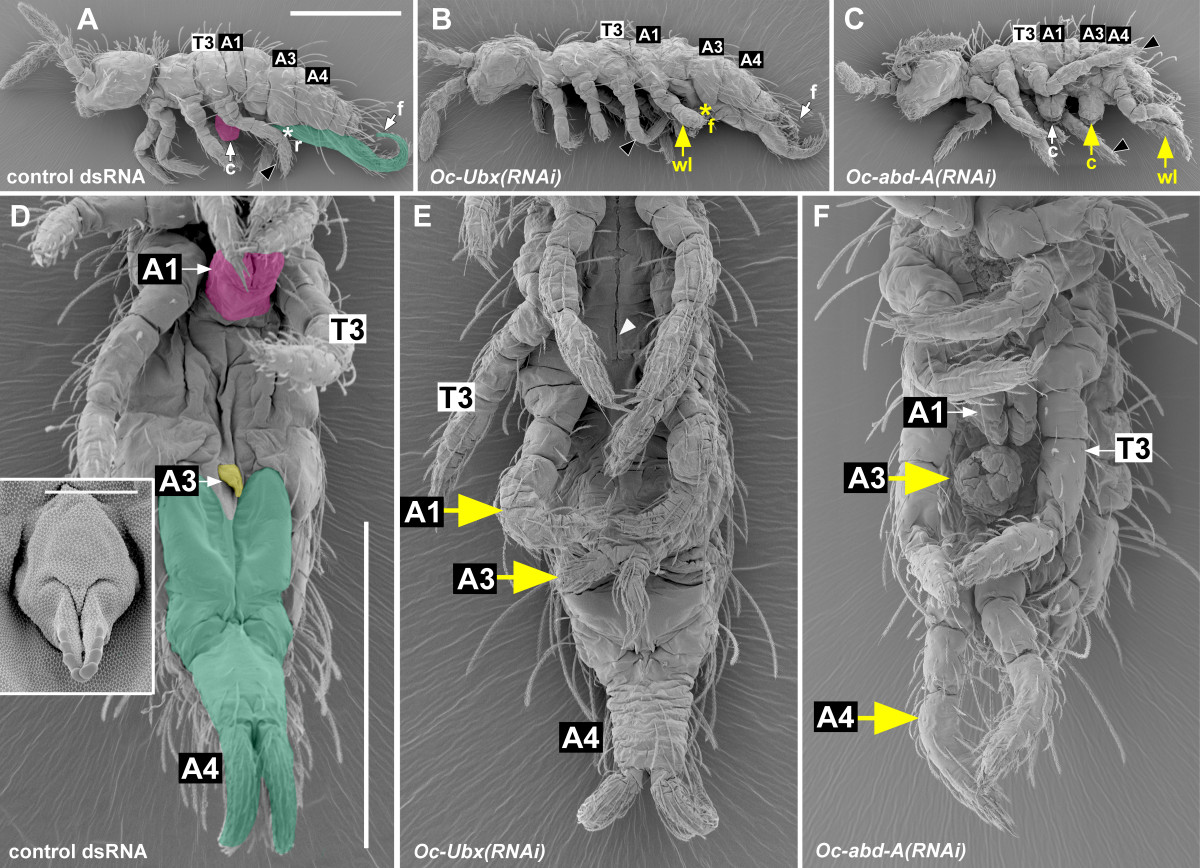
***Oc-Ubx(RNAi)*** **and** ***Oc-abd-A(RNAi)*** **lead to homeotic transformation of** ***Orchesella*** **abdominal appendages.** All panels show first instar *Orchesella* larvae, whose mothers were injected with dsRNAs. **(A-C)** lateral views, head on the left; **(D-F)** ventral views, anterior to the top. **(A, D)** Control *RNAi*. The larvae look normal. They have three pairs of thoracic legs, which are all visible in **(A)**, a collophore on A1, a retinaculum on A3, and a furca on A4; the retinaculum is hidden in **(A)**, but visible and enlarged in the inset in **(D)**. In both **(A)** and **(D)** the collophore is false coloured in magenta, the retinaculum in yellow and the furca in green. **(B, E)** *Oc-Ubx(RNAi)* larvae are abnormal in that the collophore is replaced by a pair of walking legs on A1 and the retinaculum is replaced by a furca on A3; the homeotic furca is hidden in **(B)**, but visible in **(E)**. **(C, F)** *Oc-abd-A(RNAi)* larvae are abnormal in that they have a collophore on A3 and legs on A4. The homeotically transformed appendages are labeled in yellow in all pictures. T3, A1, A3 and A4 in **(A-C)** mark the third thoracic, the first, the third and the fourth abdominal segments, respectively; in **(D-F)** their appendages. The white arrowhead in **(E)** points to the ventral groove. c, collophore; f, furca; r, retinaculum; wl, walking leg. Scale bars: in **(A)**, 200 μm for **(A-C)**; in **(D)**, 200 μm for **(D-F)**; inset, 10 μm.

The abdomen of springtails comprises six segments. Three of these bear appendages, each with a unique identity [5, 6]. These appendages arise as paired bilateral buds on A1, A3 and A4, but in late embryos each pair fuses at the ventral midline to form a specialized organ: the collophore (also called the ventral tube) on A1 participates in water absorption [7–9]. It is linked by a groove in the cuticle (the ventral groove, *linea ventralis*) to the labial glands, which probably function as excretory organs; together, these organs may function as a bipartite 'kidney’ reducing water loss through excretion [10, 11]. The retinaculum on A3 functions as a clip for the furca, which is a powerful structure formed by the appendages on A4 that is used for jumping (Figure 1). Based on morphological and embryological observations, it has been proposed that the fused proximal parts of the abdominal appendages are homologous with the coxopodites and the distal parts, which do not fuse, are homologous to the telopodites of other arthropod appendages [6].

In general, the distinct identities of segments in insects are specified by *Hox* genes [12]: in the absence of *Hox* gene function, all segments of the trunk develop similarly (reviewed in [13]). The *Hox* genes encode transcription factors that modulate many aspects of segment development, by interacting with a large number of downstream targets [14]. They are expressed from head to tail in partially overlapping domains, but typically, the more posteriorly expressed gene dominates phenotypically over the more anterior one. Changes in the domains of expression of these genes, and changes in the set of downstream targets that they regulate, have been shown to play a significant role in the evolution of arthropod diversity [15–24].

In insects, the distinct identities of different segments in the pre-genital abdomen are specified by the *Hox* genes *Ultrabithorax* (*Ubx*) and *abdominal-A* (*abd-A*) (reviewed in [25, 26]), and by *Abdominal-B,* which plays a major role in the genital segments, and a minor role more anteriorly [27–29]. We do not consider *Abd-B* further here.

In *Drosophila*, and most other insects studied, *Ubx* is initially expressed within A1 (strictly speaking, from the parasegment 5/6 boundary back) while *abd-A* is expressed from A2 (parasegment 6/7 boundary) back [26, 30–33]. It is relatively well understood how this early *Ubx* and *abd-A* expression represses limbs in *Drosophila*[34, 35]. Slightly later in development, *Ubx* expression extends anteriorly into the thorax. This later expression is unable to repress limb development, but it modifies the identity of the limbs that form [22, 35, 36].

While both *Ubx* and *abd-A* suppress appendages in *Drosophila*, in the beetle *Tribolium*, and probably also in many other less derived insects, *Ubx* does not repress appendage development in A1. Instead, it specifies on A1 the development of a pair of glandular appendages called pleuropods, which mature in the embryo and are shed at or before hatching [37, 38]. Knockdown of *Ubx* in embryos results in homeotic transformation of the pleuropods towards the phenotype of a walking leg.

How *Ubx* and *abd-A* might function to specify the multiple distinct abdominal appendages of basal hexapods is not known. An antibody that detects both Ubx and Abd-A was used by Palopoli and Patel [39] to show that either one or both of these proteins is present in each of the abdominal appendages of the springtails *Folsomia* and *Xenylla*, consistent with the hypothesis that these proteins play some role in appendage specification, but this reagent cannot discriminate between the two Hox proteins.

Despite their phylogenetic position at the base of the Hexapoda, no springtail species has been established for wide use in comparative developmental genetics, perhaps because most species are very small, the development of the larger species is often slow, and in many cases their embryos are either inaccessible or difficult to work with [40, 41]. The twin aims of our research were therefore: (1) to find a springtail that would be amenable for developmental and functional genetic experiments, and (2) to find out how *Ubx* and *abd-A* specify collembolan abdominal appendages.

Here we present our study on *Ubx* and *abd-A* function by parental RNAi in the springtail *Orchesella cincta* (Entomobryomorpha). *Orchesella* is a surface-dwelling soil springtail that reaches about 4 mm in size. It has sexual reproduction. It has previously been used for ecotoxicological experiments [42], and its genome has recently been sequenced (D. Roelofs, personal communication). We have generated an embryonic transcriptome. We show here that *Orchesella* can be raised on cultured algae and yeast as a food source and that the embryos are accessible for *in-situ* hybridization, albeit with some difficulty. We also show that parental RNAi is simple and effective for gene knockdown in this species. Using this technique, we show that both *Oc-Ubx* and *Oc-abd-A* have unique limb modulatory functions: *Oc-Ubx* on its own specifies the collophore, while *Oc-abd-A* on its own specifies the furca. Both *Oc-Ubx* and *Oc-abd-A* jointly specify the retinaculum.

## Methods

### *Orchesella cincta* culture

A culture of the springtail of *Orchesella cincta* was obtained from the Vrije Universiteit Amsterdam (kindly provided by Nico van Straalen, Dick Roelofs and Janine Mariën). Springtails were kept at 25°C with a photoperiod of 18 h light and 6 h dark in Petri dishes on a solid base made from plaster of Paris mixed with charcoal, covered with a layer of the alga *Pleurococcus* sp. (obtained from CCAP: the Culture Collection of Algae and Protozoa, Argyll, Scotland, UK) and a little baker’s yeast to serve as food. The cultures were moistened with algal liquid culture twice a week. Algae were grown in conical flasks with 3 N-BBM + V medium (recipe from CCAP website, http://www.ccap.ac.uk) standing on a windowsill. To prepare the dishes for springtails, the grown algal culture was poured onto Petri dishes with a solid base. Algae were left to settle for a few days, after which time, the liquid medium was poured out and soaked up with a piece of tissue.

### Gene cloning

Genes were isolated from cDNA prepared from mixed stages of embryos by using the ThermoScript reverse transcriptase (Invitrogen, Carlsbad, CA). The fragment of *Oc-Ubx* used for RNAi was obtained by semi-nested PCR with the Ex Taq DNA polymerase (Takara, Japan) and degenerate primers. All other isolations were done with the Phusion DNA polymerase (Finnzymes, Finland). Gene-specific primers were designed on the basis of sequences recovered in an embryonic transcriptome (Specialist Sequencing and Bioinformatics Services were provided by the EASIH, University of Cambridge). The primers are listed in (Table 1). The fragments were cloned into the pCR 4Blunt-TOPO vector (Invitrogen, Carlsbad, CA). The sequences were aligned in the CLC Sequence Viewer 6 program. Possible phosphorylation sites were identified using the NetPhos Server (http://www.cbs.dtu.dk/services/NetPhos/ and http://www.cbs.dtu.dk/services/NetPhosK/).

**Table 1:** **Primers for cloning**

Fragment	Primers (5′-3′)	
	Forward	Reverse
*Oc-Ubx* RNAi	ATGAAYTCBTAYTTYGANCA	TTCATICKICKRTTYTGRAACCA
		ATYTTDATYTGICKYTCIGTIARRCA^a^
*Oc-Ubx* probe	ACATGTATGTATTTTTCACCCTT	AGATGTATATTTTAGGTAAAGTTAC
*Oc-Ubx* full-length protein	TCAATGAACTAATTTAGGAACAATAC	AGATGTATATTTTAGGTAAAGTTAC
*Oc-abd-A* RNAi and probe	TTGGCAATCAGTGTGGTCAGG	ATATACCACACCAAGCCCACA
*Oc-abd-A* full-length protein	TTAGGGAATACAGAGGGTTCT	ATATACCACACCAAGCCCACA

^a^Nested primer in the second PCR.

### *In-situ* hybridization

Females lay eggs individually on the surface of the culture media. Eggs were collected from the Petri dishes using a fine soft paintbrush, fixed overnight in 4% formaldehyde in PBS + 0.1% Tween 20 and stored in methanol at -20°C. Eggshells (the blastodermic cuticles) [43] were manually removed using forceps. Samples were processed according to a published protocol [44] with the modification that PBS + 0.1% Tween 20 was used for washes and hybridization was at 55°C. The probe lengths were 1,251 bases for *Oc-Ubx* and 1,230 bases for *Oc-abd-A*.

### Parental RNAi

dsRNAs at lengths of 725 bases for *Oc-Ubx* and 1,230 bases for *Oc-abd-A* were synthesized using the T3 and T7 Megascript kits (Ambion, Austin, TX). *Orchesella* females were anesthetized on a carbon dioxide plate and injected with dsRNAs at a concentration of 5 μg/μl until the abdomen was obviously inflated. After an overnight recovery, the females were kept together with males of a similar age. To check that the RNAi lowered levels of the targeted mRNAs, we performed *in-situ* hybridization on the *Oc-Ubx(RNAi)* and *Oc-abd-A(RNAi)* embryos and compared the intensity of staining with that in control embryos (Additional file 1). We detected a substantial reduction in staining intensity.

### Microscopy

Samples for expression analyses were observed on a Zeiss Axioskop2 MOT plus compound microscope with a Zeiss AxioCam MRm camera, a Zeiss Axiophot compound microscope with Leica DFC300FX camera and a Leica TCS SP5 confocal microscope. For scanning electron microscopy, larvae were fixed in 80% ethanol, post-fixed with osmium tetroxide, dehydrated through an ethanol series, critical point dried, gold coated and observed on a FEI/Philips XL30 FEGSEM microscope. The photos were adjusted using Adobe Photoshop (version CS5) and Fiji.

## Results

### Laboratory culture of *Orchesella cincta*

In Amsterdam, the source culture of *Orchesella* was maintained on twigs from trees overgrown by algae and sterilized by freezing (J. Mariën, personal communication). The algae and fungi on the twigs are a vital source of food [45, 46]. Because this method does not enable quick and easy inspection of the culture and timed egg collections, we developed a method of culturing on Petri dishes with defined media. In our culturing method we feed *Orchesella* the alga *Pleurococcus* (syn. *Desmococcus*), because this alga was previously found to be that most consumed by *Orchesella* in its natural habitat. Baker’s yeast forms the fungal component in the diet. All individuals in the dishes can easily be inspected, anesthetized by filling the dish with CO_2_, and tapped into a fresh dish.

### Sequences of the *Orchesella Ubx* and *abd-A* genes

No full-length Hox protein sequences have previously been reported for springtails, though short *Hox* fragments have previously been isolated from the springtail *Folsomia candida.* We isolated cDNAs for the full protein-coding regions of *Ubx* and *abd-A* from embryos of *Orchesella*. Both genes contain the expected conserved sequence signatures, which include the homeodomain (HD), hexapeptide motif (HX) and UbdA peptide (Additional file 2 and Additional file 3). Two isoforms were recovered for both *Oc-Ubx* and *Oc-abd-A*. The isoforms differ in the length of the linker region (LR) between the hexapeptide and the homeodomain (8 vs 12 amino acids in Oc-Ubx; 27 vs 49 in Oc-abd-A). The linker in the long isoform of Oc-abd-A is noticeably long compared with that in other arthropod abd-A sequences.

A common feature of insect Ubx sequences is the presence of two motifs C-terminal to the UbdA peptide; the QAQA motif and the poly-alanine stretch, which were both shown to be important for the limb repressive role of Ubx in insects [47, 48]. The QAQA motif, but not the poly-alanine stretch, was found in *Folsomia* Ubx. Similarly, Oc-Ubx contains the QAQA motif, but lacks the poly-alanine stretch suggesting that this is a common feature of springtail Ubx. The C-termini of Ubx from diverse arthropods, but not the insects, contain several phosphorylation sites; the phosphorylation at these sites was shown to block the general ability of Ubx to repress appendages [48]. The C-terminus of *Folsomia* Ubx contains one phosphorylation site. We did not find any predicted phosphorylation sites in Oc-Ubx C-terminal to the UbdA peptide, suggesting that the single site in *Folsomia* Ubx is not a common feature of springtail Ubx. The C-termini of the insect abd-A sequences are typically enriched in the amino acid glutamine, but we did not find any glutamine residues in the C-terminus of Oc-abd-A. The TD motif, which is a site for interaction with cofactors, and is present in insect but not other arthropod abd-A sequences [49], is missing in Oc-abd-A.

In summary, the Oc-Ubx sequence shows a combination of ancestral and novel (insect) features, while the Oc-abd-A is more similar to abd-A sequences from arthropods other than insects.

### Embryonic expression of *Oc-Ubx* and *Oc-abd-A*

**Figure 2:**
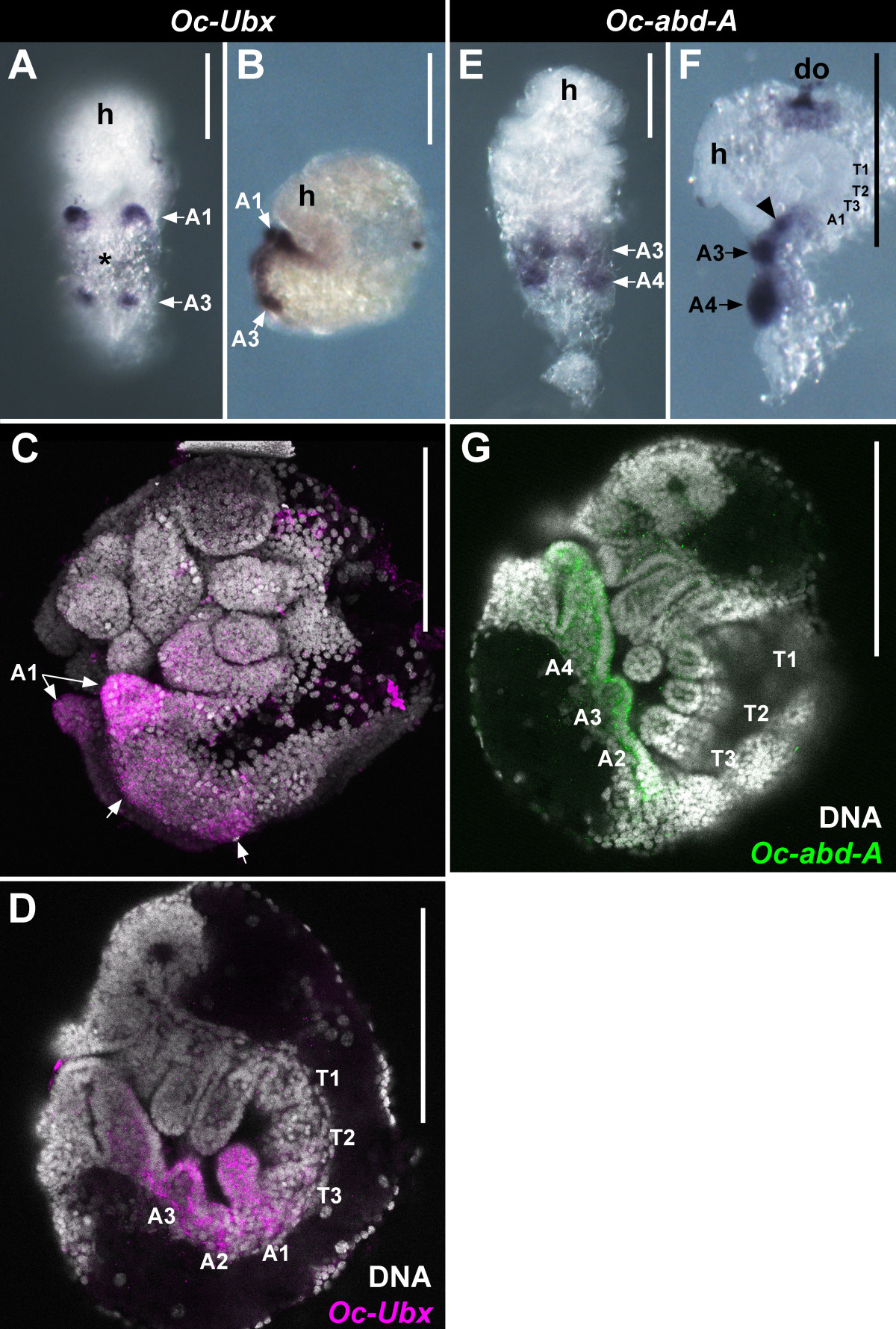
**Expression of** ***Oc-Ubx*** **and** ***Oc-abd-A*** **in** ***Orchesella*** **embryos.** Bright field images of NBT/BCIP stained **(A, B, E, F)** and confocal images of Fast Red stained **(C, D, G)** *Orchesella* embryos. **(A-D)** *Oc-Ubx* expression and **(E-G)** *Oc-abd-A* expression. **(A and E)** are ventral views, other pictures are lateral views, head at the top. *Orchesella* embryogenesis lasts 4.75 days; the embryos in **(A, C, E)** are 30 h, in **(B, F)** 32 h, and in **(D, G)** 48 h old. **(A)** The early signs of *Oc-Ubx* expression are seen as two pairs of dots (A1, A3), with a weak smudge in the middle (asterisk). **(B)** In an older embryo, the staining is more intense, extended laterally and slightly anteriorly. The staining does not spread behind A3. **(C)** Confocal image of an embryo aged as the embryo in **(A)**. The intensity of staining obtained using this technique is stronger. In all **(A-C)** the expression is highest in A1. **(D)** In an older embryo with well-developed abdominal appendages, *Oc-Ubx* is detected in A1-A3. **(E)** The expression of *Oc-abd-A* appears as two pairs of dots (A3, A4). **(F)** In a slightly older embryo the staining is more intense and spreads one segment (black arrowhead) anteriorly. Staining in the dorsal organ (do) is not specific. **(G)** In an embryo with well-developed abdominal appendages, *Oc-abd-A* is detected in A2 to A4. A1 to A4, the first to the fourth abdominal segment; do, dorsal organ; h, head; T1 to T3, the first to the third thoracic segment. The interpretation of appendage identity is based on the observation of dozens of embryos; abdominal appendages can be distinguished by their specific morphology. Scale bars: all 100 μm.

To distinguish between the expression domains of *Oc-Ubx* and *Oc-abd-A* in *Orchesella* embryos, we studied the distribution of their transcripts by *in-situ* hybridization. Springtail embryos are not easy to handle because they are small, they are tightly bent during the critical developmental stages and they are surrounded by highly refractile yolk. We combined NBT/BCIP staining and bright field microscopy, which provides low background, but also relatively poor resolution, with Fast Red staining and confocal microscopy, which provides higher resolution, but also, in our hands, high background (Figure 2). The specific shape of the A1 appendage buds served as a marker for distinguishing between abdominal segments in young embryos.

In early stages of expression both *Oc-Ubx* and *Oc-abd-A* were detected as two pairs of spots, which we identified as buds on A1 and A3 for *Oc-Ubx* and A3 and A4 for *Oc-abd-A* (Figure 2A,E). Expression of both genes was also visible in A2, but was less intense (Figure 2A,C). In slightly older embryos, the expression spread to other parts of the segments and became more intense (Figure 2B,F). In embryos with well-defined abdominal appendages, *Oc-Ubx* was evident in A1 to A3 and *Oc-abd-A* in A2 to A4 segments (Figure 2D,G). We could not see whether the expression domains extended also to the posterior part of the next most anterior segment (that is, T3p for *Oc-Ubx*; A1p for *Oc-abd-A*), as they do in many insects.

### Knockdown of *Oc-Ubx* leads to homeotic transformation of the A1 and A3 appendages

To understand the function of *Oc-Ubx* and *Oc-abd-A* in *Orchesella*, we reduced the levels of gene function in embryos by injecting double-stranded RNA for each gene into the abdomens of female parents (a process colloquially known as 'knock down by parental RNAi’) [50]. In *Tribolium*, and an increasing number of other insect species tested, this has been shown to reduce gene function, though there is considerable variability in both the degree and duration of knockdown for different genes, and in different species [51, 52].

As a first step, we injected two types of control dsRNA targeted against sequences that were not expected to be present in Orchesella; the *egfp* gene of jellyfish and the *MalE* gene of E. coli. Neither of these dsRNAs interfered with normal development of the females or their progeny (Table 2, Figure 1A,D).

**Table 2:** **Efficiency of** ***Oc-Ubx*** **and** ***Oc-abd-A*** **silencing by parental RNAi in** ***Orchesella***

dsRNA	Females		Larvae collected	Homeotic phenotype		
	Injected	Survived		None	Weak	Strong
*egfp*	96	80 (83%)	415	415 (100%)	-	-
*MalE*	27	20 (74%)	195	195 (100%)	-	-
*Oc-Ubx*	77	52 (67.5%)	249	58 (23.5%)	44 (17.5%)	147 (59%)
*Oc-abd-A*	99	67 (68%)	622	140 (22.5%)	205 (33%)	277 (44.5%)
*Oc-Ubx* + *MalE*	27	12 (44%)	98	41 (42%)	15 (15%)	42 (43%)
*Oc-abd-A* + *MalE*	42	26 (62%)	173	75 (43.5%)	32 (18.5%)	66 (38%)
*Oc-Ubx* + *Oc-abd-A* (injected once)	49	37 (76%)	141	7 (5%)	107 (76%)	27 (19%)
*Oc-Ubx* + *Oc-abd-A* (injected twice)	102	47 (46%)	43	4 (9.5%)	6 (14%)	33 (76.5%)

Eggs were collected for 10 days after female injections. All larvae hatched; we did not count the unfertilized eggs, which are occasionally laid by RNAi, control and non-injected females and always represent less than 1%. dsRNAs for *egfp* and *MalE* genes served as controls. The table shows only a part of our experiments where we scored and counted all the larvae. In total, we observed offspring from 239, 99, 316, 120 females injected with *egfp*, *MaleE*, *Oc-Ubx* and *Oc-abd-A* dsRNA, respectively; we always saw only the phenotypes specific for each treatment. Homeotic phenotypes: strong: transformed appendages morphologically similar to their normal counterparts; weak: appendages with signs of transformation, but malformed, for example, A1 and A4 partially fused proximally in *Oc-Ubx(RNAi)* or *Oc-abd-A(RNAi)*, A3 blister-like in *Oc-abd-A(RNAi)*, elongated, non-segmented, partially fused proximally in *Oc-Ubx + Oc-abd-A(RNAi)*.

Larvae from the females injected by *Oc-Ubx* dsRNA also hatched normally, but 76.5% of the larvae that hatched from eggs collected within the first 10 days after the injections died at the end of the first instar and showed homeotic transformation of the A1 and A3 appendages (Table 2). The collophore on A1 was transformed into a pair of walking legs and the retinaculum on A3 into a structure resembling the furca (Figure 1B,E).

**Figure 3:**
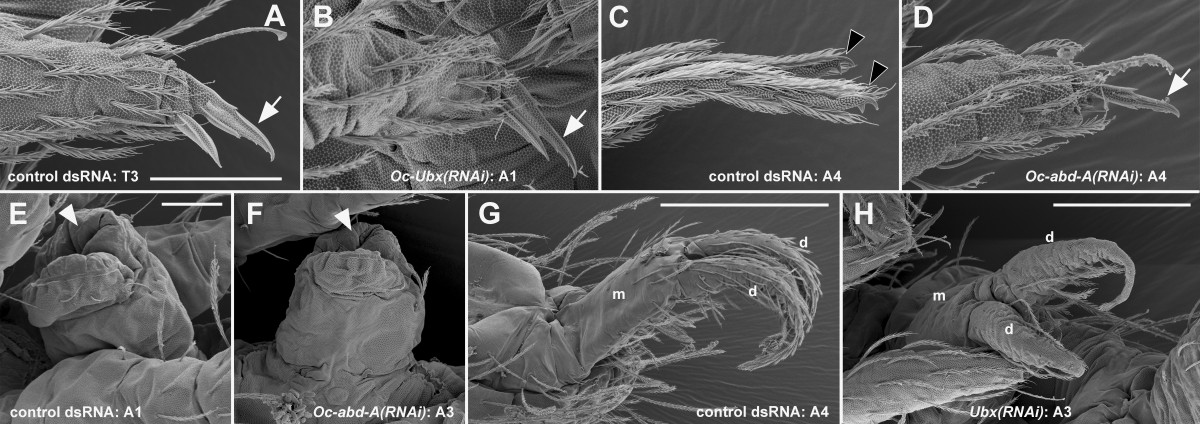
**Details of the homeotically transformed appendages. A-D**. The homeotic legs on A1 **(B)** and A4 **(D)** have a terminal claw like the control walking legs on T3 **(A)**; the claws are marked with white arrows. Note the difference between **(B)** and the control collophore on A1 in **(E)**, and between **(D)** and the tip of the control furca on A4 in **(C)**. The two appendages forming the furca terminate with specialized structures (mucrones), marked here with black arrowheads; both appendages are shown. **(E, F)** The collophore on A3 **(F)** resembles the control collophore on A1 by its gross morphology and the presence of characteristic smooth cuticle at its tip (white arrowheads). **(G, H)** The homeotic furca on A3 has, like the control furca on A4, the proximal common manubrium (m) bifurcated into two distal dentes (d), although it is smaller (compare the scale bars). Most of the setae on the homeotic furca in **(H)** were lost during sample preparation. Note how the A3 homeotic appendages in **(F)** and **(H)** differ from the control retinaculum on A3 in (Figure 1D). Scale bars: in A, 20 μm for **(A-D)**; in E, 20 μm for **(E, F)**; **(G)** 100 μm; **(F)** 50 μm.

**Figure 4:**
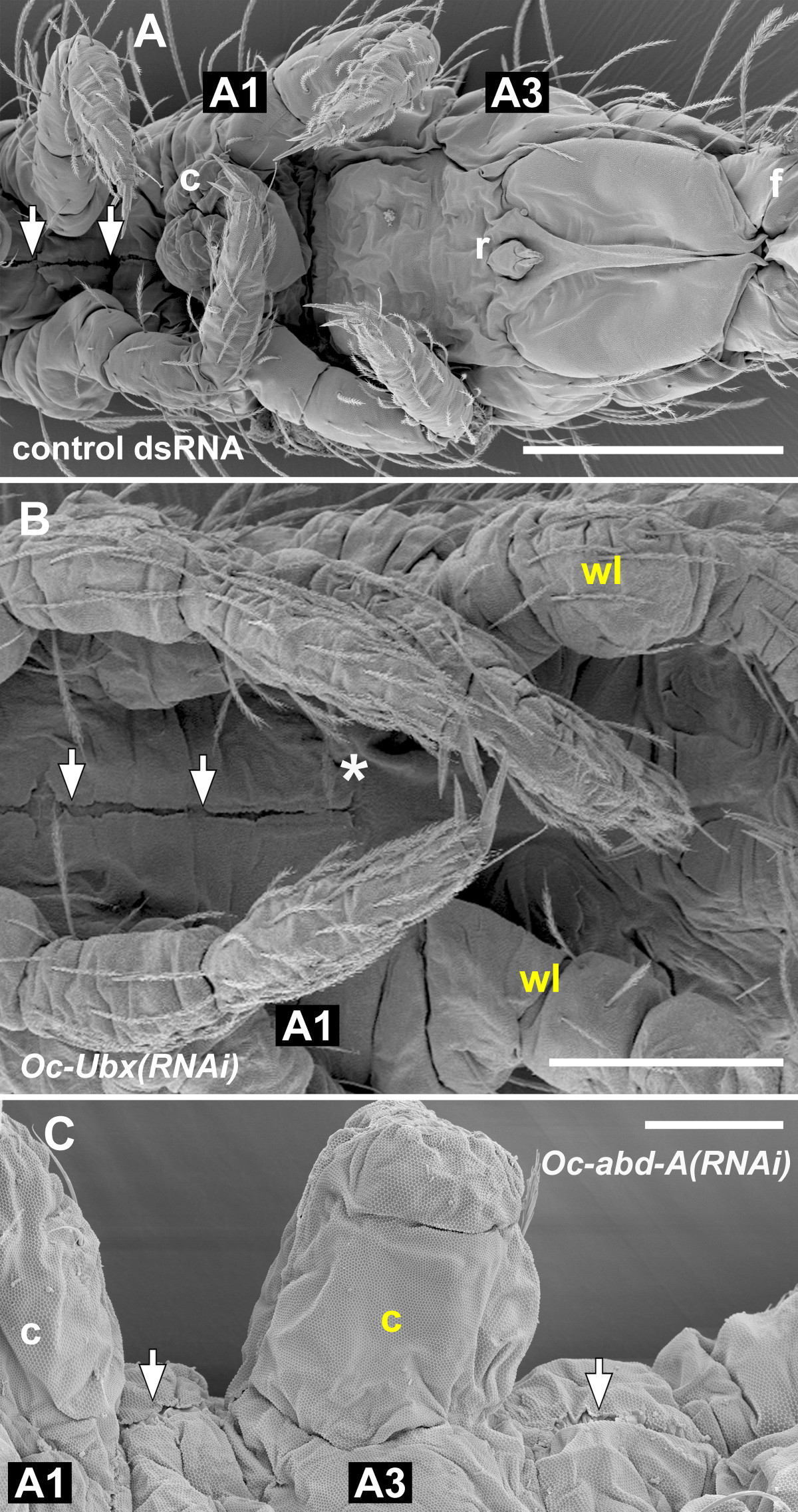
***Oc-abd-A*** **inhibits the ventral groove. (A)** In wildtype (and control) embryos, the ventral groove (white arrows) is a channel in the cuticle that runs on the ventral side of the thorax from the head to the tip of the collophore (c) on A1 and never extends behind. **(B)** In the *Oc-Ubx(RNAi)* larvae, which have a pair of walking legs on A1 instead of the collophore, the ventral groove (white arrows) terminates between the homeotic walking legs (asterisk). **(C)** In the *Oc-abd-A(RNAi)* larvae, the ventral groove (white arrows) leads to the homeotic collophore on A3 and even extends behind. A1 and A3 mark the first and the third abdominal segments, respectively. c, collophore; f, furca; r, retinaculum; wl, walking leg. The homeotic appendages are labelled in yellow. Scale bars: **(A)** 100 μm; **(B)** 50 μm; **(C)** 20 μm.

**Figure 5:**
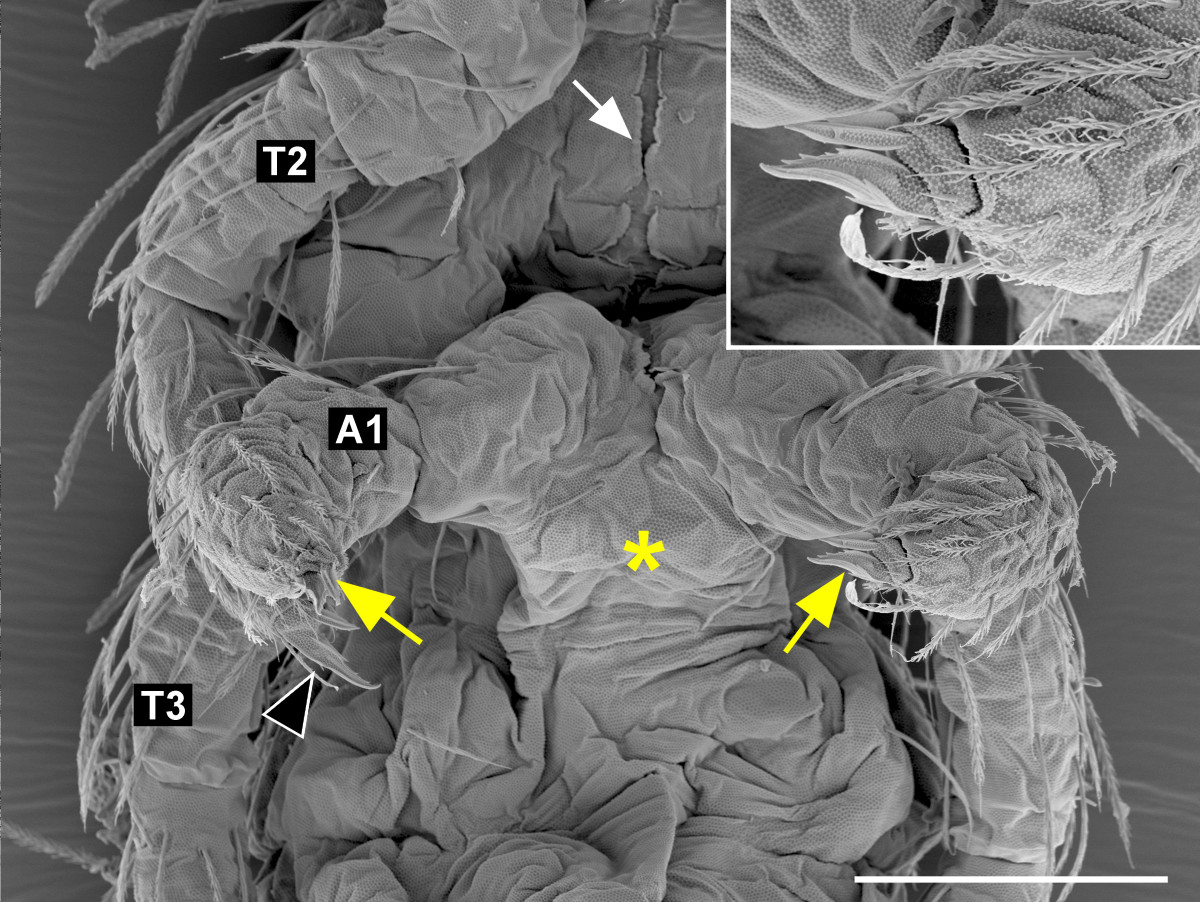
**Weak** ***Oc-Ubx(RNAi)*** **phenotype.** Low levels of *Oc-Ubx* are sufficient for appendage fusion, but do not prevent development of walking leg morphology. The appendages on A1 of this larva resemble the legs and have claws at their tips (yellow arrows and inset; compare with the claw at the tip of the thoracic leg marked with a black arrowhead), but their proximal parts are fused (asterisk). The white arrow marks the ventral groove. A1, T2, T3 mark the first abdominal, the second and the third thoracic legs, respectively. Scale bar: 50 μm.

By external observation, the ectopic pair of legs on A1 was, in the most strongly transformed individuals, identical with the thoracic legs: the proximal parts of the A1 legs were separate (Figure 1E), unlike the fused appendages of the normal collophore, and the appendages ended with a claw (Figure 3B; compare with Figure 3A). The legs moved when the larvae walked (Additional file 4). The ventral groove, which normally leads from the labial segment to the tip of the collophore, ended in between the A1 legs (Figure 1E, Figure 4B; compare with Figure 4A). In the less strongly transformed individuals, the legs on A1 were fused in the midline while still retaining their leg-like identity (Figure 5); this suggests that low levels of *Oc-Ubx* are sufficient to promote appendage fusion, but higher levels are required to transform the legs into the vesicular organ of the collophore.

Additional file 4: **The** ***Oc-Ubx(RNAi)*** **larvae move with their homeotic legs on A1 when they walk** **.** The movie shows a larva slowly walking by using all four pairs of legs. The black arrow marks the A1 leg, the outlined arrows mark the thoracic legs. The first pair of legs is less visible, because it is kept just under the head. These larvae have problems with walking because of the voluminous furca on A3, which is the homeotically transformed retinaculum. As they do not have the retinaculum (the 'holder’ of the furca), the natural furca on A4 is not attached to the ventral side as normally, but hangs down and is usually dragged behind. The object in the front is an empty eggshell. The movie was captured by Olympus μ digital camera pointed at a computer screen projecting pictures from a Leica MZIII stereomicroscope adapted with a Leica DFC500 camera. (MOV 9 MB)

The ectopic furca on A3 resembled the normal furca on A4, except that even in the most strongly transformed individuals it was smaller (Figure 1E, Figure 3H; compare with Figure 3G). In summary, *Oc-Ubx* in *Orchesella* is required for giving the appendage buds on A1 the collophore identity and those on A3 the retinaculum identity; a general function of *Oc-Ubx* is to promote the fusion of paired appendage primordia.

### Knockdown of *Oc-abd-A* leads to homeotic transformation of the A3 and A4 appendages

We next repeated the injections with *Oc-abd-A* dsRNA. As with *Oc-Ubx(RNAi)*, injection of *Oc-abd-A(RNAi)* had no effect on hatching. In over 75 per cent of the resulting larvae, the retinaculum on A3 was transformed into a collophore and the furca on A4 was transformed into walking legs (Table 2). Strong transformations are illustrated in (Figure 1C and F). These larvae fed and walked until the end of the first instar, when most of them died. Only a few of the more weakly affected individuals started ecdysis to the second instar; these died during shedding the cuticle or soon after.

The collophore on A3 of the most strongly transformed individuals was similar in morphology and size to the endogenous collophore on A1 (Figure 1C,F; compare with Figure 1D; Figure 3F; compare with Figure 3E). The ectopic legs on A4 were segmented like legs; their proximal parts were separate (Figure 1C,F) and they had a claw at their tips (Figure 3D; compare with Figure 3A,C). They were not moved during walking. The ventral groove was extended from its normal ending at the collophore in A1 throughout A2 to the tip of the ectopic collophore on A3 and even behind it (Figure 4C; compare with Figure 4A,B); his suggests that in normal animals one role of *Oc-abd-A* is to inhibit development of the ventral groove in segments posterior to A1.

In summary, *Oc-abd-A* is required for specification of the retinaculum on A3 and the furca on A4. It induces fusion of the appendages (similarly to *Oc-Ubx*) and in the cuticle it represses the ventral groove. In the absence of *Oc-abd-A*, both the A3 and the A4 appendages transform to a more anterior fate.

### Simultaneous knockdown *of Oc-Ubx* and *Oc-abd-A* transforms all *Orchesella* abdominal appendages into leg-like structures but does not induce appendages on A2

The experiments above show that when *Orchesella* abdominal appendages are missing the function of both *Oc-Ubx* and *Oc-abd-A* they have a walking leg identity. We observed this in the A1 appendages in *Oc-Ubx(RNAi)* and A4 appendages in *Oc-abd-A(RNAi)*. We next wanted to test whether simultaneous silencing of *Oc-Ubx* and *Oc-abd-A* by double RNAi would transform all *Orchesella* abdominal appendages into legs, and whether it would allow the development of legs on A2, which normally makes no appendage.

Effective knockdown of two genes is difficult, because the maximum amount of each type of dsRNA that can be injected is reduced to one half compared with the single-gene RNAi. To probe the strength of double RNAi in *Orchesella*, we first co-injected mothers with dsRNA against one of the *Hox* genes mixed with control dsRNA. The penetrance of the phenotype was slightly reduced for both *Oc-Ubx* and *Oc-abd-A*, but the strong transformation was still observed in more than 38 percent of larvae (Table 2).

**Figure 6:**
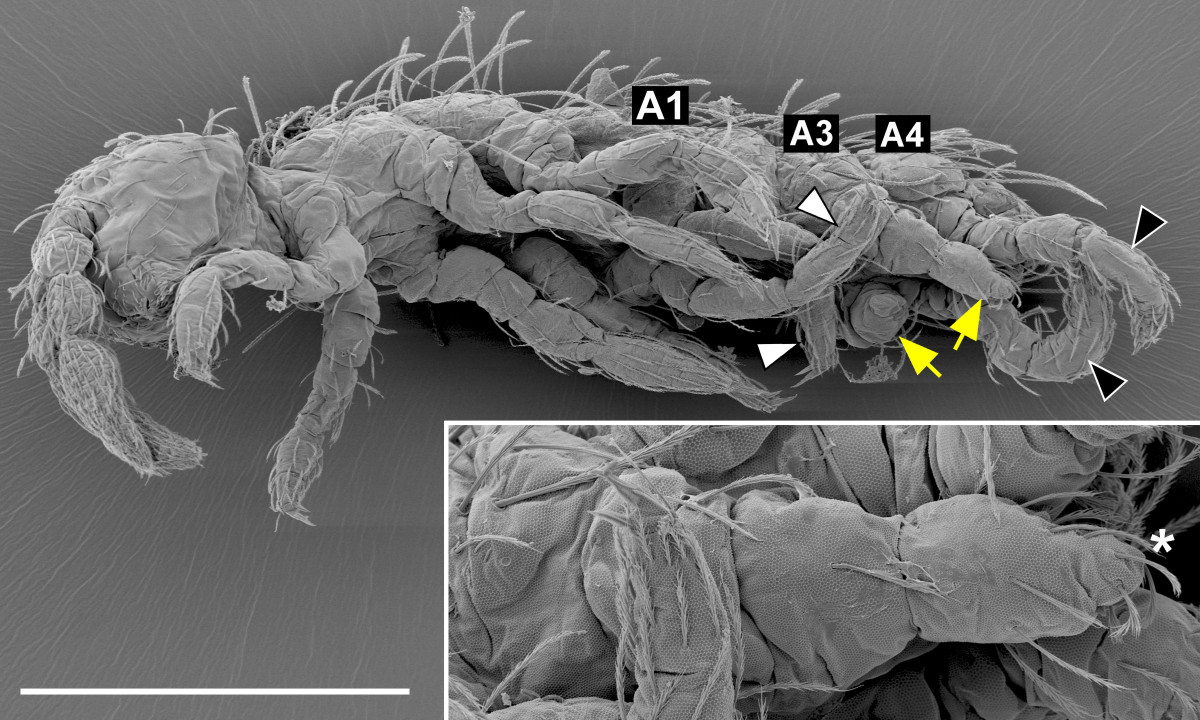
**Double** ***Oc-Ubx + Oc-abd-A(RNAi)*** **phenotype.** Larva that had both *Oc-Ubx* and *Oc-abd-A* knocked down by parental RNAi has legs on A1 (white arrowheads) and A4 (black arrowheads) as in the single-gene RNAi. The A3 appendages (yellow arrows) also resemble legs, but are missing terminal parts. This A3 'leg’ is enlarged in the inset; the asterisk marks the distal tip lacking the claw. A1, A3 and A4 mark the first, the third and the fourth abdominal segments, respectively. Scale bar: 200 μm.

When we injected the females with both *Oc-Ubx* and *Oc-abd-A* dsRNAs together, some of their progeny had all of their abdominal appendages transformed towards the leg identity (Figure 6). The frequency of the strongest phenotypes was higher when the maternal injections were repeated on two successive days (19 per cent after one versus 76.5 per cent after two injections) but the number of eggs laid by the twice-injected females was lower. More than two consecutive injections led to lethality in the females without production of eggs. In the most strongly transformed individuals, the legs on A1 and A4 were identical with those that we observed in the single-gene RNAi. The appendages on A3 were segmented like legs and had their proximal parts separated, but remained short and lacked claws (Figure 6, inset). No appendages developed on A2.

In summary, these experiments show that double RNAi is possible in *Orchesella*, because we achieved a novel transformation compared with single-gene RNAi.

## Discussion

**Figure 7:**
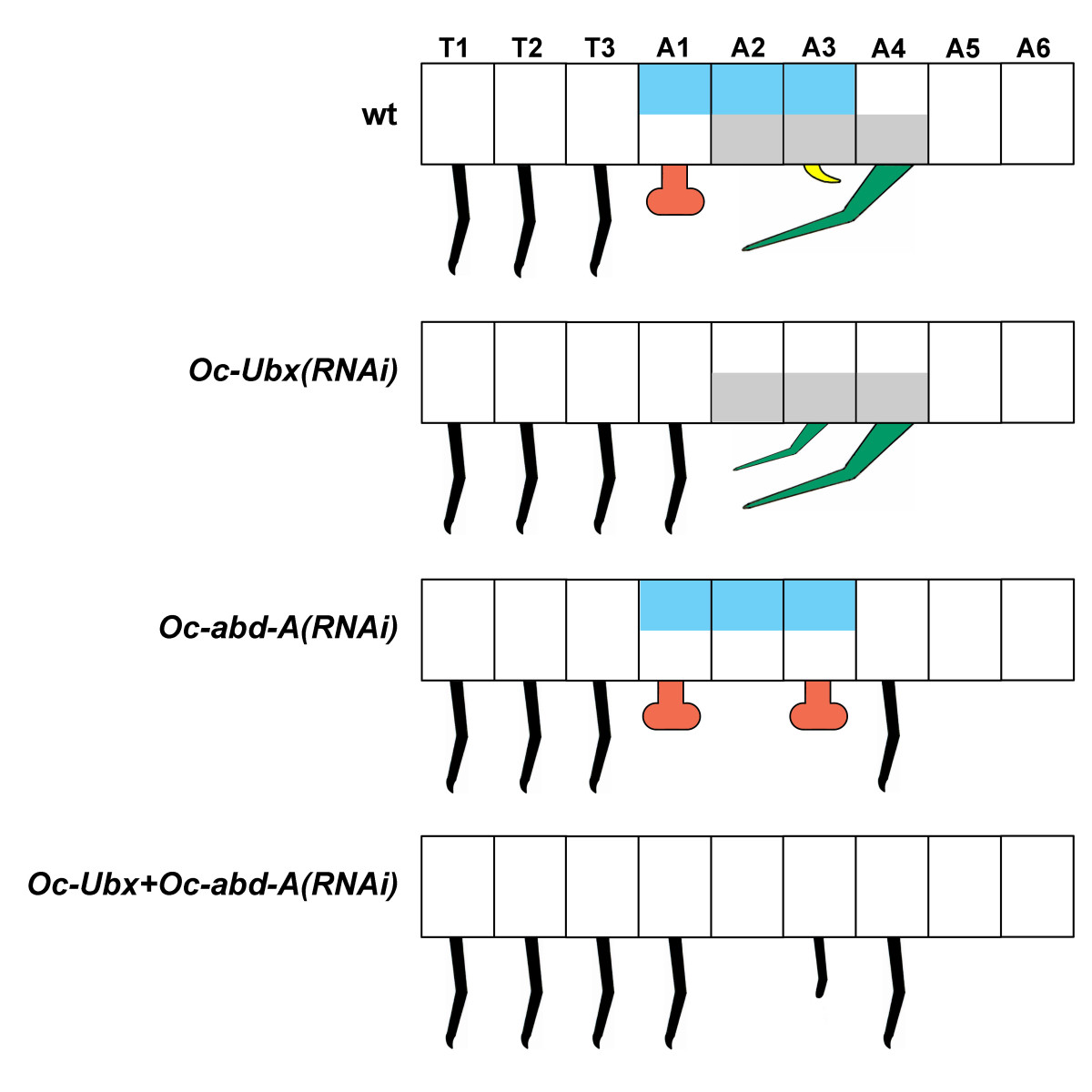
**Schematic summary of** ***Oc-Ubx*** **and** ***Oc-abd-A*** **knockdown experiments in** ***Orchesella*** **.** The boxes represent the thoracic (T1 to T3) and abdominal (A1 to A6) segments. Blue and grey shading represent *Oc-Ubx* expression and *Oc-abd-A* expression, respectively (examined by *in-situ* hybridization on wild type, control and knockdown embryos). The appendage buds in which only *Oc-Ubx* is expressed develop into the collophore, the buds in which only *Oc-abd-A* is expressed develop into the furca, and the buds in which both *Oc-Ubx* and *Oc-abd-A* are expressed develop into the retinaculum. When neither *Oc-Ubx* nor *Oc-abd-A* is expressed, the appendage has a leg identity. The appendages on A3 are smaller, such as the furca in *Oc-abd-A(RNAi)*, and may be missing distal parts, such as the 'legs’ in the double *Oc-Ubx + Oc-abd-A(RNAi)*. The A2 segment never develops appendage buds. wt, wildtype.

In this study we have investigated the role of the *Hox* genes *Ubx* and *abd-A* in the specification of abdominal appendages in the springtail *Orchesella*. The results of our RNAi experiments are summarized diagrammatically in Figure 7. We conclude that:

- The activity of *Oc-Ubx* alone causes appendage buds to develop as a collophore, because (i) when *Oc-Ubx* is knocked down, the buds on A1, which express *Oc-Ubx* but not *Oc-abd-A*, develop into legs, not a collophore; (ii) when *Oc-abd-A* is knocked down, the buds on A3, which normally express both *Oc-Ubx* and *Oc-abd-A*, develop into a collophore, presumably because depletion of *Oc-abd-A* leaves *Oc-Ubx* alone expressed in these buds.
- The activity of *Oc-abd-A* alone causes appendage buds to develop as a furca, because (i) when *Oc-abd-A* is knocked down, the buds on A4, which express only *Oc-abd-A*, develop into legs, not the furca; (ii) when *Oc-Ubx* is knocked down, so that presumably *Oc-abd-A* alone is expressed in A3, the A3 buds develop into a furca.
- If both *Oc-Ubx* and *Oc-abd-A* are expressed in a pair of limb buds, it develops as a retinaculum.

### *Oc-Ubx* specifies a unique identity for A1 in hexapods

In collembolans, as in insects, A1 is the only abdominal segment that is specified by *Ubx* in the absence of *abd-A*. Silencing of *Oc-Ubx* in *Orchesella* replaces the collophore with a pair of walking legs. Similarly in insects, depletion of *Ubx* alone results in the appearance of leg structures on A1 [12, 26, 38, 53, 54]. In insects, in the absence of *Ubx*, expression of the more anterior *Hox* gene *Antennapedia* persists in A1, where it is normally repressed by *Ubx*[55], resulting in the development of a leg-like appendage. It is likely that the same happens in collembolans, although this has not been tested.

In dipterans and some other endopterygote insects, for example, the lepidopteran *Manduca*[56], *Ubx* functions as a limb repressor, and A1 bears no appendages. In many other species though, including the beetle *Tribolium*, the milkweed bug *Oncopeltus* and the cricket *Acheta*, *Ubx* specifies the embryonic glandular appendages on A1 called pleuropods [26, 37, 38, 53]. Like the collophore of springtails, pleuropods are vesicular structures that are likely involved in secretion or excretion, but unlike the collophore, they remain paired rather than forming a single midline organ, and they degenerate before hatching [57, 58]. It seems possible that the specification of this derived type of glandular appendage is a conserved ancestral role of *Ubx* in hexapods, and that the collophore in collembolans is a homologue of the insect pleuropods. *Ubx* presumably targets a unique set of genes in A1 to specify these organs. These targets have presumably been lost in *Drosophila*, concomitant with the acquisition of a limb suppression role, but they may have been conserved throughout most of hexapod evolution, as pleuropods are present in some advanced lineages of Endopterygota, such as Lepidoptera and Coleoptera.

### *Oc-abd-A* does not repress appendage development in springtails

We argue that the role of *Oc-abd-A* expressed alone in the limb buds of *Orchesella* is to specify appendage buds to develop as the furca, and that in combination with *Oc-Ubx* it specifies the development of the retinaculum. It has been shown that the middle section of the furca expresses the gene *dac*[41], which is normally expressed in the middle part of the thoracic leg [59]; this suggests that the furca is homologous to the full-length leg. Thus, *Oc- abd-A* does not repress formation of any part of the leg, but modifies it. This is quite different from the general role of *abd-A* in the pterygote insects, such as *Drosophila*, *Tribolium* and *Oncopeltus*, where *abd-A* represses appendage bud formation from an early stage in development, by repressing the expression of the *distal-less (dll)* gene [26, 34, 60, 61]. The observation that the *Orchesella abd-A* sequence lacks several of the derived features seen in insects is consistent with this very different role.

There is a precedent for *abd-A* promoting specific appendage development in pterygote insects. In the larvae of the silkworm *Bombyx*, *abd-A* specifies the abdominal locomotory appendages known as prolegs, present on A3 to A6 [62, 63]. However, parsimony suggests that this is a secondary adaptation that evolved long after *abd-A* acquired a role in limb repression.

It is probably premature to speculate whether limb repression or appendage specification is the ancestral role of *abd-A* in hexapods until further studies of appendage specification are carried out in basal hexapods and apterygote insects. In Diplura, and in the primitively wingless insects Archaeognatha (jumping bristletails) and Zygentoma (silverfish), abdominal segments up to A9 may develop small appendages called styli and ventral sacs (for embryogenesis see, for example, [64]). The fossil record has shown that abdominal styli were also present in the adults of Palaeozoic pterygotes [65]. It is clear, though, from studies of crustaceans and spiders [24, 66] that *abd-A* specifies unique appendage types in other arthropod groups, for example, in crustaceans that have distinct appendages on the pereon and pleon.

### Combinatorial specification of the A3 appendage by both *Oc-Ubx* and *Oc-abd-A*

The limb buds on A3 express both *Oc-Ubx* and *Oc-abd-A*. Both are required for the development of a morphologically and functionally unique appendage, the retinaculum [5]. Interestingly, when *Oc-abd-A* is removed, the A3 appendage acquires a more anterior identity (collophore), as is commonly the case in *Hox* knockdowns, but when *Oc-Ubx* is removed, the A3 appendage acquires a more posterior identity; that of the furca. In this case, the more posterior *Hox* gene expressed in A3, *Oc-abd-A*, does not show posterior prevalence [67, 68], but acts combinatorially with *Oc-Ubx* to specify the A3 identity.

It remains to be seen whether the combinatorial regulation of A3 appendage morphology by *Oc-Ubx* and *Oc-abd-A* is indeed a true combinatorial role of these two Hox proteins functioning together in individual limb bud cells, or whether distinct cell populations in the bud express one or the other of these two proteins at different times, and the combinatorial function is at the supracellular level. The limited resolution of our *in-situ* analysis cannot distinguish between these possibilities. This would require specific antibodies for Oc-Ubx and Oc-abd-A.

### Why do no appendages develop on A2?

All body segments of arthropods have the potential to develop appendages. If appendages are absent in the segment, it is generally because they are suppressed by a *Hox* gene, which frequently acts by repressing *dll* expression, a developmental gene that acts in the early stages of limb initiation. The regulation of *dll* may either be direct or indirect, for example, via *btd*[34, 59, 69].

Springtails never have appendages on A2, and in those species tested, this segment does not express *dll*[39]. We might therefore expect that one of the *Hox* genes expressed in the abdomen represses limb development in A2.

We have shown in *Orchesella* that both *Oc-Ubx* and *Oc-abd-A* are expressed in A2, although the expression is at lower levels, and perhaps initiated later, than in other segments. However, neither *Oc-Ubx* nor *Oc-abd-A* seems to repress limbs, because silencing either or both of these genes, sufficiently to transform other limb identities, never leads to the development of appendages on A2. What does suppress appendage primordia in A2 remains unknown.

One possibility is that a specific limb repressing *Hox* gene is expressed in A2. We have no evidence that other copies of *Oc-Ubx* or *Oc-abd-A* exist in the collembolan genome. No other copies are present in our embryonic transcriptome, and none was identified in an extensive screen of *Folsomia Hox* fragments by PCR. It is possible that *Abd-B* plays a role in A2, because in some arthropods its expression domain extends anteriorly (for example, [70, 71]). We consider this less likely, because *Abd-B* typically specifies the genital segments and in *Orchesella* the genital opening is localized between segments A5 and A6. This remains to be tested.

Alternatively, the development of limb buds on A2 might be suppressed not by a *Hox* gene, but by some upstream factor in the segmentation hierarchy, analogous to the gap genes in *Drosophila*. Such factors distinguish different regions along the anteroposterior axis before and during segment specification (reviewed in [72]). There is no reason *a priori* why they should not locally regulate *dll* or other early expressed limb specification factors, though we are aware of no precedents for such effects.

A single specimen of a collembolan having a retinaculum on A2 has been found in nature (cited in [5]). Clearly therefore, there is some gene whose malfunction can lead to the development of an appendage on A2. The fact that this appendage developed as a retinaculum is consistent with our observation that A2 normally expresses both *Oc-Ubx* and *Oc-abd-A*, as does the normal A3, which develops the retinaculum.

### The role of *Oc-Ubx* and *Oc-abd-A* genes in structures other than the appendage primordia

Apart from its role in specification of the appendages, our results clearly show that *Oc-abd-A* has a role in the ventral epidermis, where it normally represses the ventral groove posterior to the collophore, on A2, A3 and A4.

It is possible that other ectodermal structures are modified by *Oc-Ubx* and *Oc-abd-A* knockdown, and therefore that whole segments are homeotically transformed. Apart from the ventral groove, however, there are no obvious characteristics of the cuticle morphology that are segment specific in the abdomen, and so might reveal these roles. We have not examined in detail either the size of cuticular regions in the different segments, or the precise distribution of bristles and other sensillae in the cuticle that might reveal such effects.

### *Orchesella* as a springtail model

Because of their small size, springtails are often overlooked, but they are widespread and abundant soil-living organisms. They play an important role in soil decomposition and serve as models for ecotoxicological research [5, 73, 74]. Compared with other springtails *Orchesella* has several features that make it well suited for genetic experiments. It is conveniently large (for a springtail: 4 mm!), it has a relatively short generation time (1 month at 25°C), and it can be kept continuously in laboratory conditions. *Orchesella* is a surface-dwelling springtail adapted to heterogeneous moisture conditions and so is relatively resistant against desiccation [75, 76]. In natural conditions, *Orchesella* suffers from high mortality by predation, which is compensated by high fecundity [77]; in the culture, about 30 eggs are laid by a female every 2 to 3 days.

The homeotic transformations presented in this paper demonstrate that gene knockdown by parental RNAi functions well in *Orchesella*. It is likely that RNAi will work equally well for many other genes, allowing functional studies of both development and physiology. The current disadvantage of *Orchesella* is that the early embryonic stages (before the appendage buds are visible) are not accessible, because a tough egg cuticle is secreted at a very early stage of development. This problem applies to other collembolans that have been used for embryological studies [41]. However, we hope that a way will be found to circumvent it.

## Conclusions

The springtail *Hox* genes *Ubx* and *abd-A* each specify a distinct appendage type when acting on their own; together, they specify a third, novel, appendage identity.

RNAi functions well in *Orchesella*; the phylogenetic position of this species at the base of the Hexapoda, its ease of culture in the laboratory and the available genetic resources, suggest that it will be of further use for comparative genetic studies.

## Electronic supplementary material

Additional file 1: **Parental RNAi in** ***Orchesella*** **lowers endogenous mRNA levels in the offspring.** Embryos aged 48 hours that were laid by females injected with either control (*egfp*) dsRNA **(A,C)**, *Oc-Ubx* **(B)** or *Oc-abd-A* **(D)** were hybridized with probes recognizing either *Oc-Ubx* **(A,B)** or *Oc-abd-A* **(C,D)** mRNAs and detected with NBT-BCIP staining producing dark blue colour. All samples were processed simultaneously. Embryos were photographed from the lateral side. The site of specific staining is marked by arrows in one embryo from each treatment; h marks the head. Asterisks mark non-specific staining (mostly in the dorsal organs). The staining in *RNAi* embryos is present, but it is weaker than in the controls. (JPEG 2 MB)

Additional file 2: **Amino acid alignments of** **Oc-Ubx** **sequence with related sequences from other species.** Long isoform of Oc-Ubx is shown; the underlined amino acids are missing in the short isoform. Only one out of the nine Oc-Ubx clones that we sequenced is 'long’; five 'short’ clones have alanine (A) and three clones have threonine (T) in the position 145 (arrow). The poly-alanine stretch (highlighted in blue) is missing in both springtail Ubx sequences. Accession numbers (GenBank unless otherwise specified): springtails: EMBL:HG530310 (Oc, *Orchesella cincta*), AAK51917 (Fc, *Folsomia candida*); insects: NP_536752 (Dm, *Drosophila melanogaster*), NP_001107632 (Bm, *Bombyx mori*), NP_001034497 (Tc, *Tribolium castaneum*), NP_001162171 (Am, *Apis mellifera*), AEB15973 (Of, *Oncopeltus fasciatus*); crustaceans: AAL67686 (Af, *Artemia franciscana*), ACT53742 (Ph, *Parhyale hawaiensis*); a myriapod (centipede): ABD16212 (Sm, *Strigamia maritima*); a chelicerate (spider): CAX11340 (Pt, *Parasteatoda tepidariorum*). The longest known Ubx protein sequences were used for alignments. HD, homeodomain; HX, hexapeptide motif; UbdA, UbdA peptide. Parts of the sequences that have not been isolated yet are marked with '?’. (JPEG 4 MB)

Additional file 3: **Amino acid alignments of** **Oc-abd-A** **sequence with related sequences from other species** **.** Long isoform of Oc-abd-A is shown; the underlined amino acids are missing in the short isoform. The C-terminus of Oc-abd-A does not contain any glutamine (Q) residues, while the C-termini of insect abd-A sequences are highly enriched in the amino acid glutamine (Q, highlighted in green. HD, homeodomain; HX, hexapeptide motif; UbdA, UbdA peptide. TD motif (highlighted in grey) is present in insect sequences, but missing in Oc-abd-A. UR motif [78] is dashed underlined in the *Drosophila* sequence (this domain is in the Bm, Tc and Am sequence split apart in the alignment). Accession numbers (GenBank unless otherwise specified): springtails: EMBL:HG530313 (Oc, *Orchesella cincta*), AAK52498 (Fc, *Folsomia candida*); insects: AAF55360 (Dm, *Drosophila melanogaster*), NP_001166808 (Bm, *Bombyx mori*), NP_001034518 (Tc, *Tribolium castaneum*), XP_394120 (Ap, *Apis mellifera*), XP_001944629 (Ap, *Acyrthosiphon pisum*); a crustacean: ACS36775 (Af, *Artemia franciscana*); a myriapod (centipede): ABD16213 (Sm, *Strigamia maritima*); a chelicerate (spider): CAA07502 (Cs, *Cupiennius salei*). The longest known Ubx protein sequence from each species were used for alignments. HD, homeodomain; HX, hexapeptide motif; UbdA, UbdA peptide. Parts of the sequences that have not been isolated yet are marked with '?’. (JPEG 4 MB)

Below are the links to the authors’ original submitted files for images.

Authors’ original file for figure 1

Authors’ original file for figure 2

Authors’ original file for figure 3

Authors’ original file for figure 4

Authors’ original file for figure 5

Authors’ original file for figure 6

Authors’ original file for figure 7

## Abbreviations

A1: A2, A3…: first, second, third … abdominal segments
abd-A: *Abdominal-A*
dll: *Distal-less*
HD: homeodomain
HX: hexapeptide motif
LR: linker region
PBS: phosphate-buffered saline
PCR: polymerase chain reaction
RNAi: RNA interference
Ubx: *Ultrabithorax*
T3: third thoracic segment.
