# Supplementary figures and images for: The Hox genes Ultrabithorax and abdominal-A specify three different types of abdominal appendage in the springtail Orchesella cincta (Collembola)
Source: EvoDevo. 2014 Jan 7;5:2. doi: 10.1186/2041-9139-5-2 (PMC3910676; doi:10.1186/2041-9139-5-2)

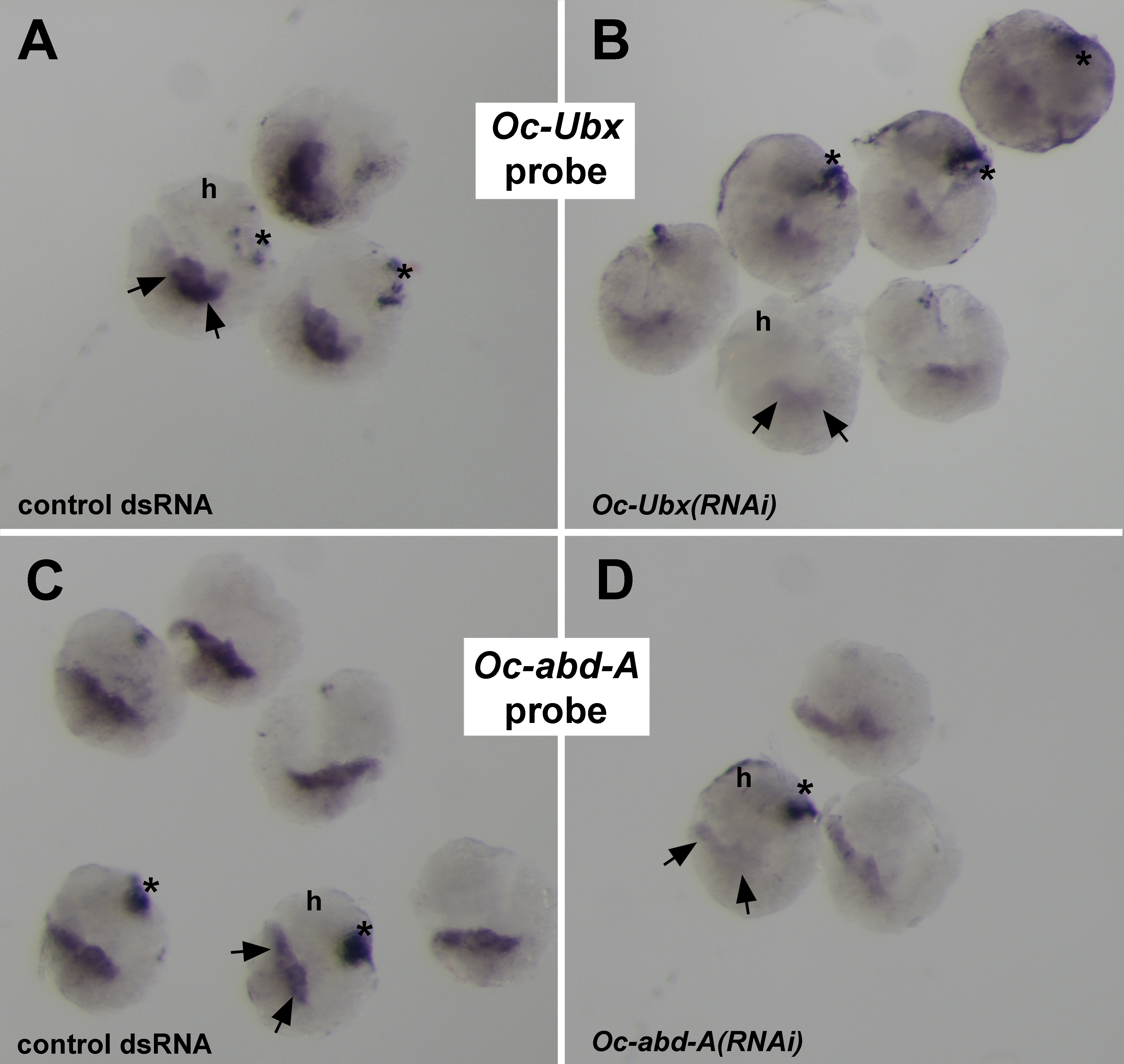

Supplement: Supplementary file 2 — Additional file 1: Parental RNAi in Orchesella lowers endogenous mRNA levels in the offspring. Embryos aged 48 hours that were laid by females injected with either control (egfp) dsRNA (A,C), Oc-Ubx (B) or Oc-abd-A (D) were hybridized with probes recognizing either Oc-Ubx (A,B) or Oc-abd-A (C,D) mRNAs and detected with NBT-BCIP staining producing dark blue colour. All samples were processed simultaneously. Embryos were photographed from the lateral side. The site of specific staining is marked by arrows in one embryo from each treatment; h marks the head. Asterisks mark non-specific staining (mostly in the dorsal organs). The staining in RNAi embryos is present, but it is weaker than in the controls. (JPEG 2 MB) [file 13227_2013_140_MOESM1_ESM.jpeg]

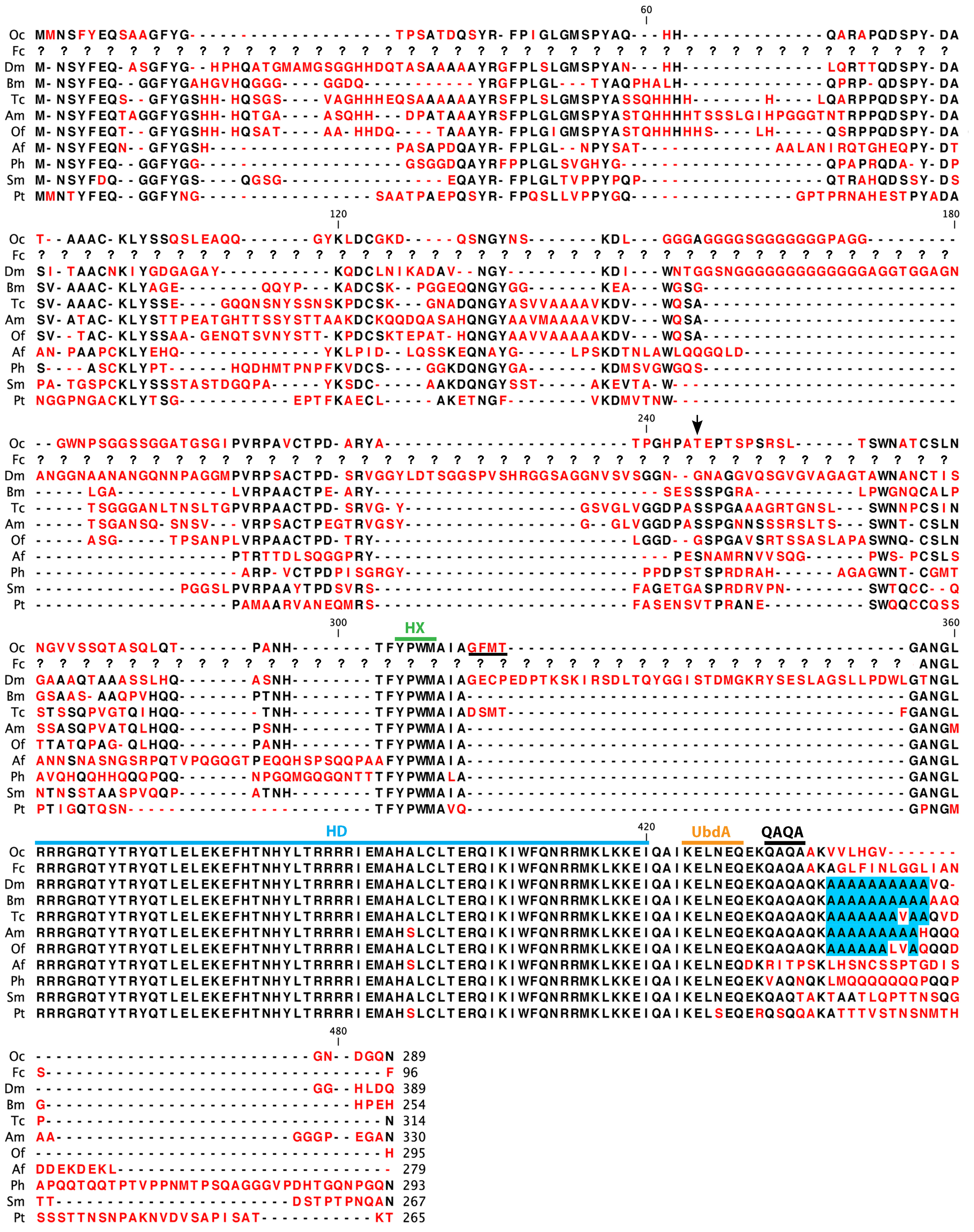

Supplement: Supplementary file 3 — Additional file 2: Amino acid alignments of Oc-Ubx sequence with related sequences from other species. Long isoform of Oc-Ubx is shown; the underlined amino acids are missing in the short isoform. Only one out of the nine Oc-Ubx clones that we sequenced is 'long’; five 'short’ clones have alanine (A) and three clones have threonine (T) in the position 145 (arrow). The poly-alanine stretch (highlighted in blue) is missing in both springtail Ubx sequences. Accession numbers (GenBank unless otherwise specified): springtails: EMBL:HG530310 (Oc, Orchesella cincta), AAK51917 (Fc, Folsomia candida); insects: NP_536752 (Dm, Drosophila melanogaster), NP_001107632 (Bm, Bombyx mori), NP_001034497 (Tc, Tribolium castaneum), NP_001162171 (Am, Apis mellifera), AEB15973 (Of, Oncopeltus fasciatus); crustaceans: AAL67686 (Af, Artemia franciscana), ACT53742 (Ph, Parhyale hawaiensis); a myriapod (centipede): ABD16212 (Sm, Strigamia maritima); a chelicerate (spider): CAX11340 (Pt, Parasteatoda tepidariorum). The longest known Ubx protein sequences were used for alignments. HD, homeodomain; HX, hexapeptide motif; UbdA, UbdA peptide. Parts of the sequences that have not been isolated yet are marked with '?’. (JPEG 4 MB) [file 13227_2013_140_MOESM2_ESM.jpeg]

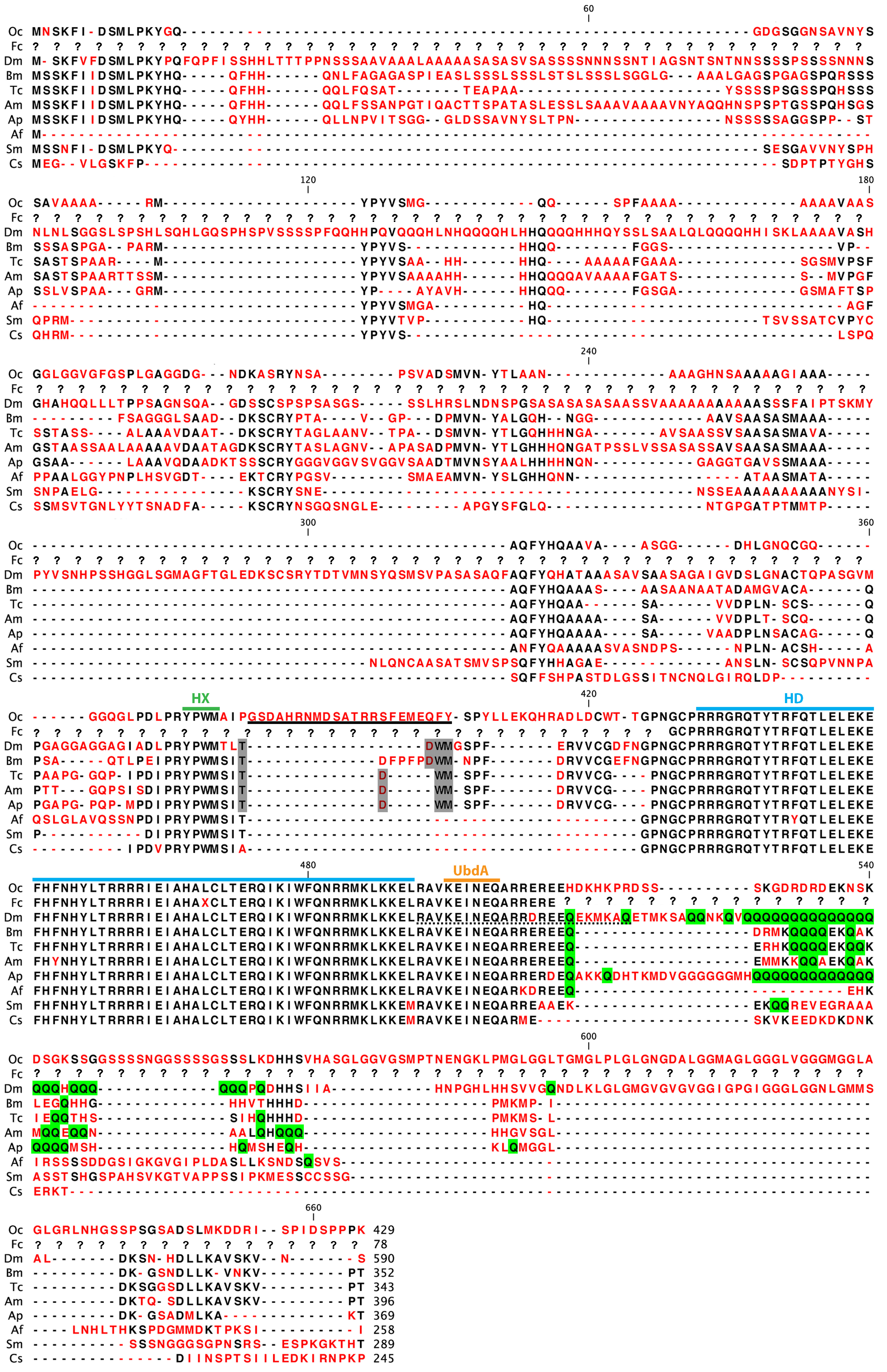

Supplement: Supplementary file 4 — Additional file 3: Amino acid alignments of Oc-abd-A sequence with related sequences from other species . Long isoform of Oc-abd-A is shown; the underlined amino acids are missing in the short isoform. The C-terminus of Oc-abd-A does not contain any glutamine (Q) residues, while the C-termini of insect abd-A sequences are highly enriched in the amino acid glutamine (Q, highlighted in green. HD, homeodomain; HX, hexapeptide motif; UbdA, UbdA peptide. TD motif (highlighted in grey) is present in insect sequences, but missing in Oc-abd-A. UR motif [78] is dashed underlined in the Drosophila sequence (this domain is in the Bm, Tc and Am sequence split apart in the alignment). Accession numbers (GenBank unless otherwise specified): springtails: EMBL:HG530313 (Oc, Orchesella cincta), AAK52498 (Fc, Folsomia candida); insects: AAF55360 (Dm, Drosophila melanogaster), NP_001166808 (Bm, Bombyx mori), NP_001034518 (Tc, Tribolium castaneum), XP_394120 (Ap, Apis mellifera), XP_001944629 (Ap, Acyrthosiphon pisum); a crustacean: ACS36775 (Af, Artemia franciscana); a myriapod (centipede): ABD16213 (Sm, Strigamia maritima); a chelicerate (spider): CAA07502 (Cs, Cupiennius salei). The longest known Ubx protein sequence from each species were used for alignments. HD, homeodomain; HX, hexapeptide motif; UbdA, UbdA peptide. Parts of the sequences that have not been isolated yet are marked with '?’. (JPEG 4 MB) [file 13227_2013_140_MOESM3_ESM.jpeg]

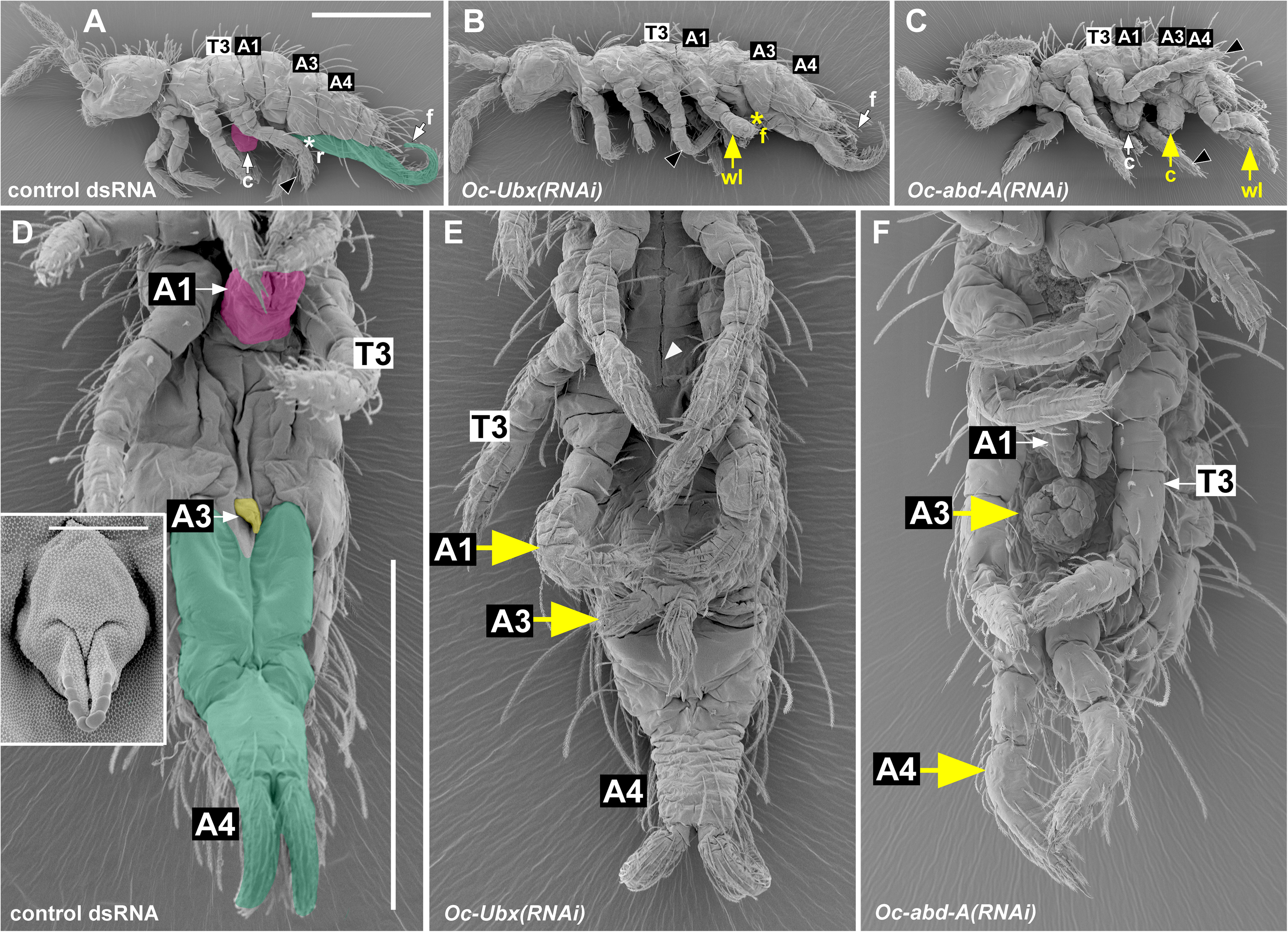

Supplement: Supplementary file 5 — Authors’ original file for figure 1 [file 13227_2013_140_MOESM5_ESM.tiff]

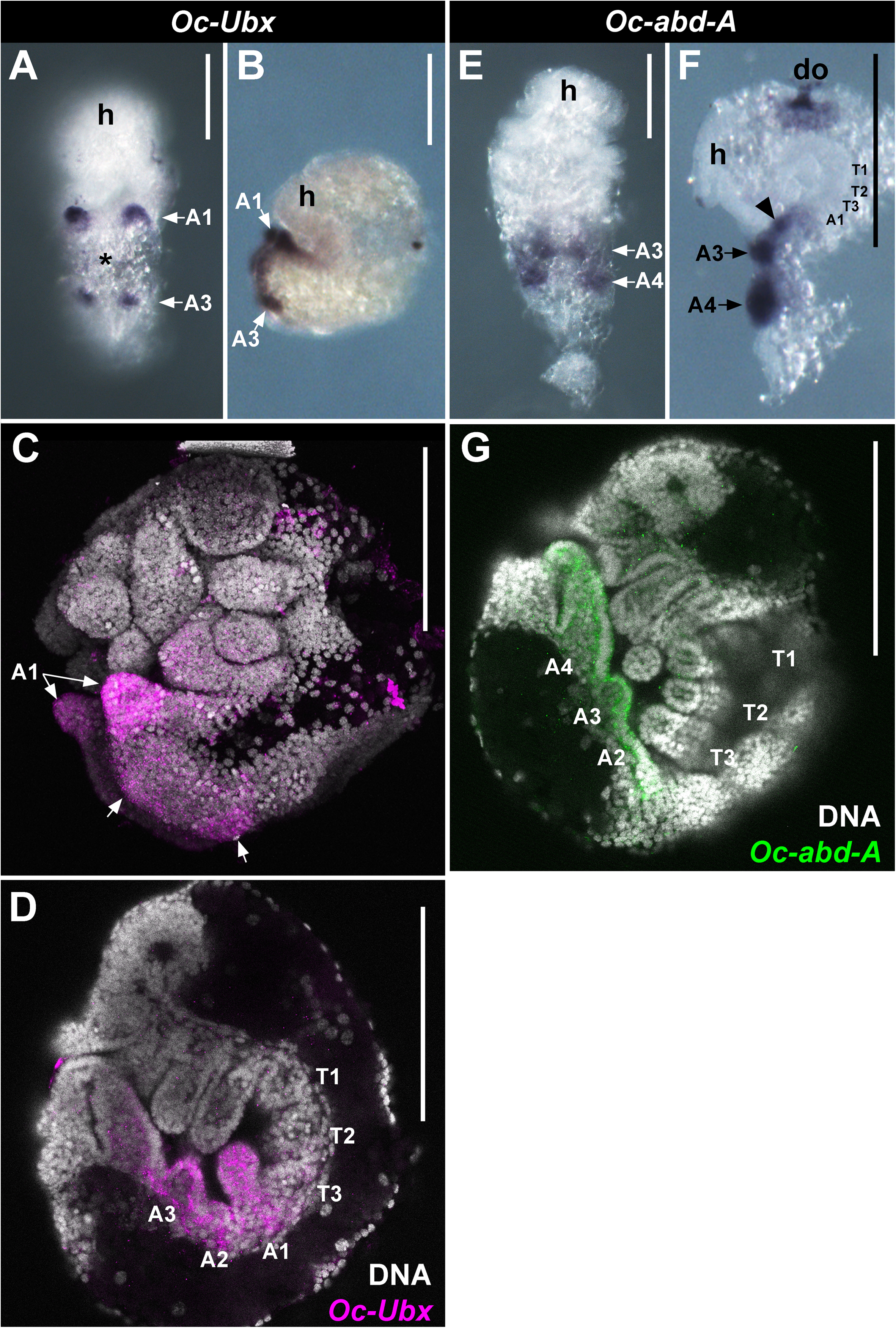

Supplement: Supplementary file 6 — Authors’ original file for figure 2 [file 13227_2013_140_MOESM6_ESM.tiff]

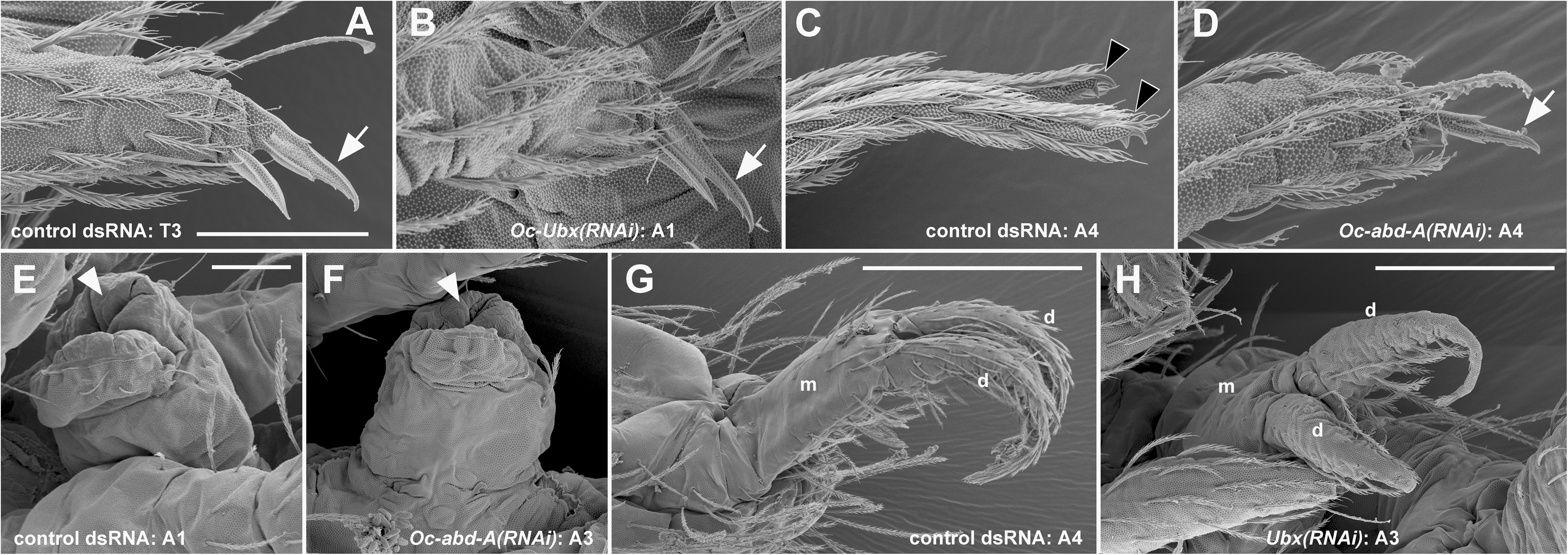

Supplement: Supplementary file 7 — Authors’ original file for figure 3 [file 13227_2013_140_MOESM7_ESM.tif]

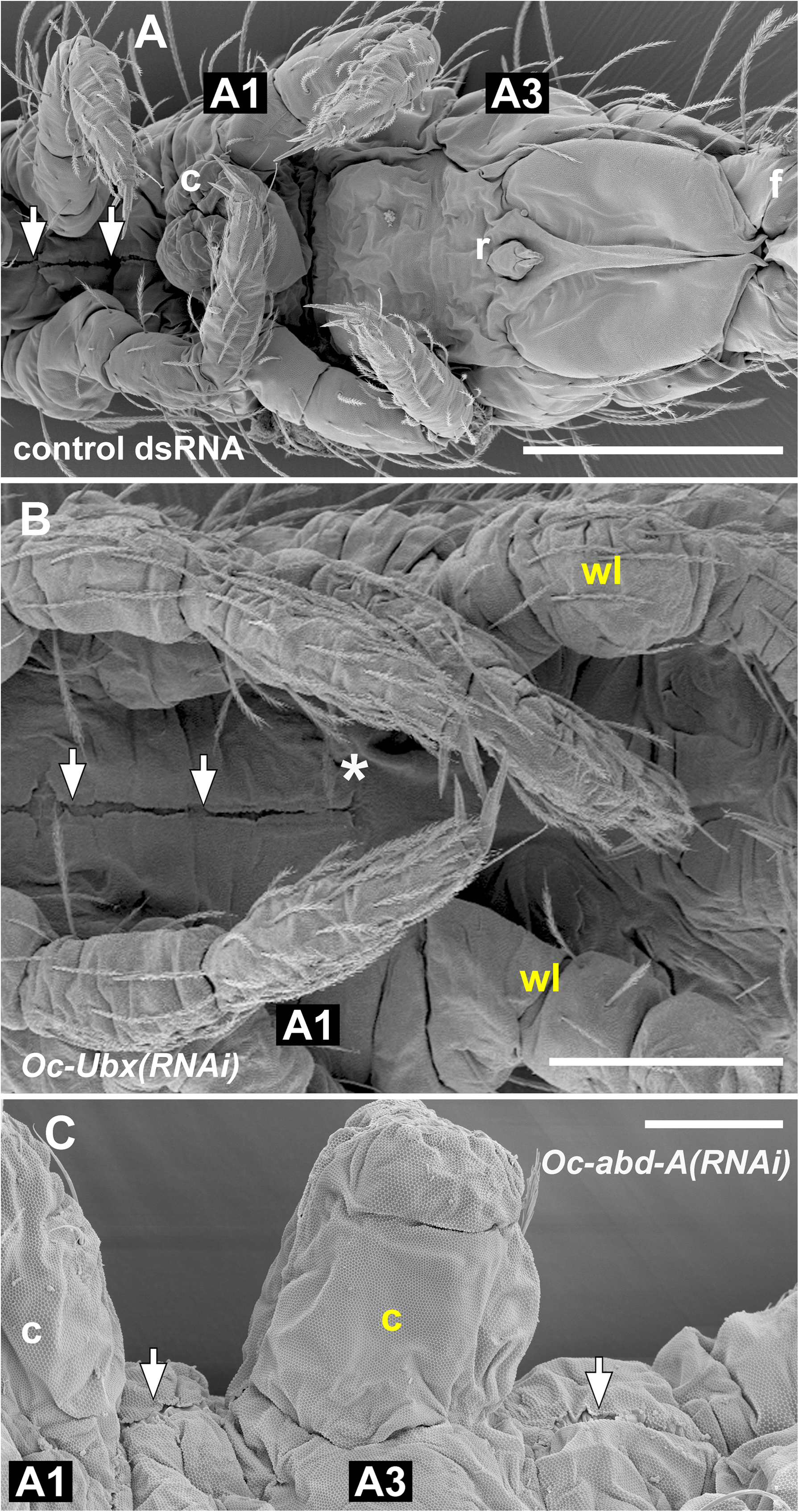

Supplement: Supplementary file 8 — Authors’ original file for figure 4 [file 13227_2013_140_MOESM8_ESM.tif]

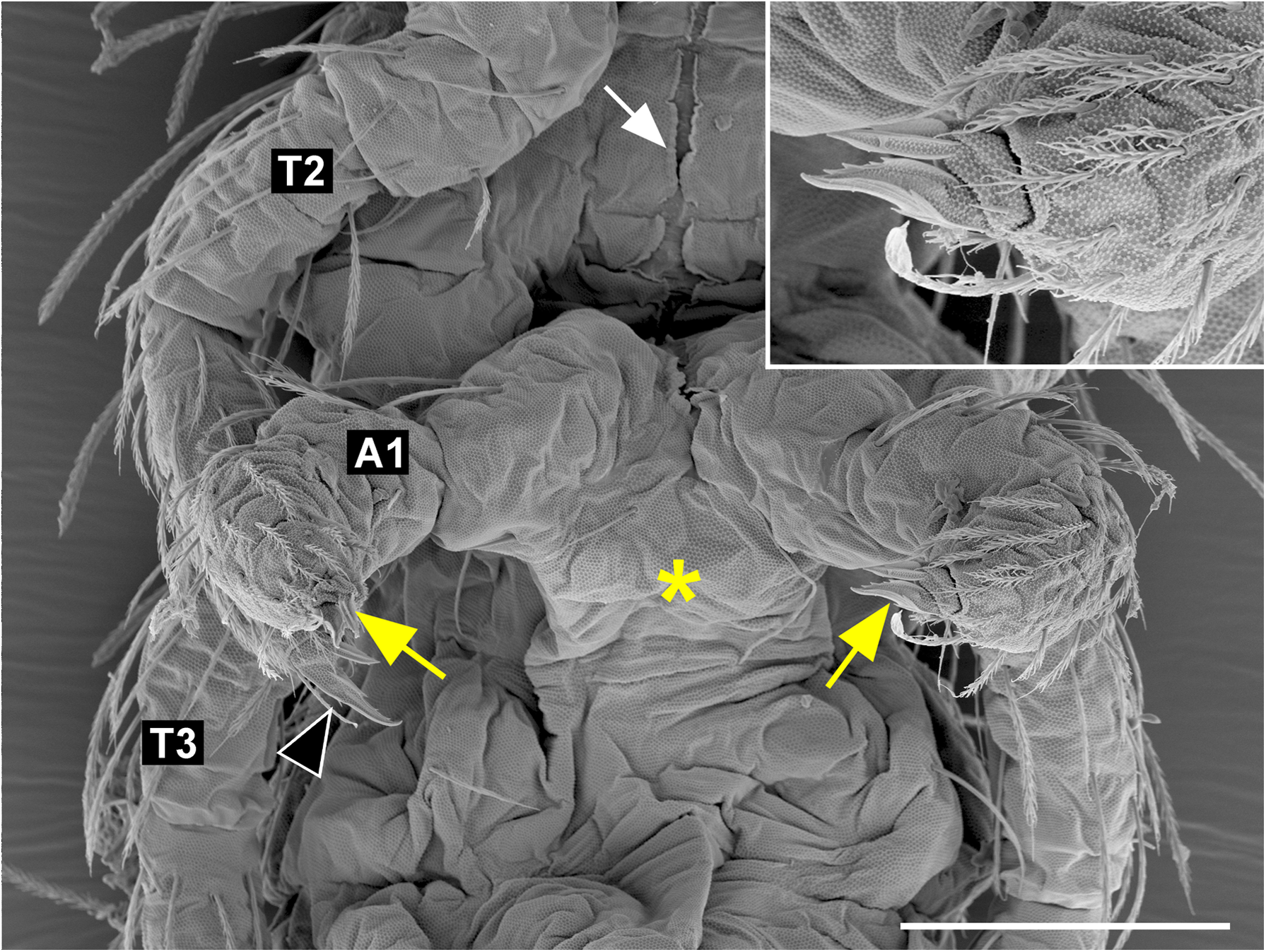

Supplement: Supplementary file 9 — Authors’ original file for figure 5 [file 13227_2013_140_MOESM9_ESM.tif]

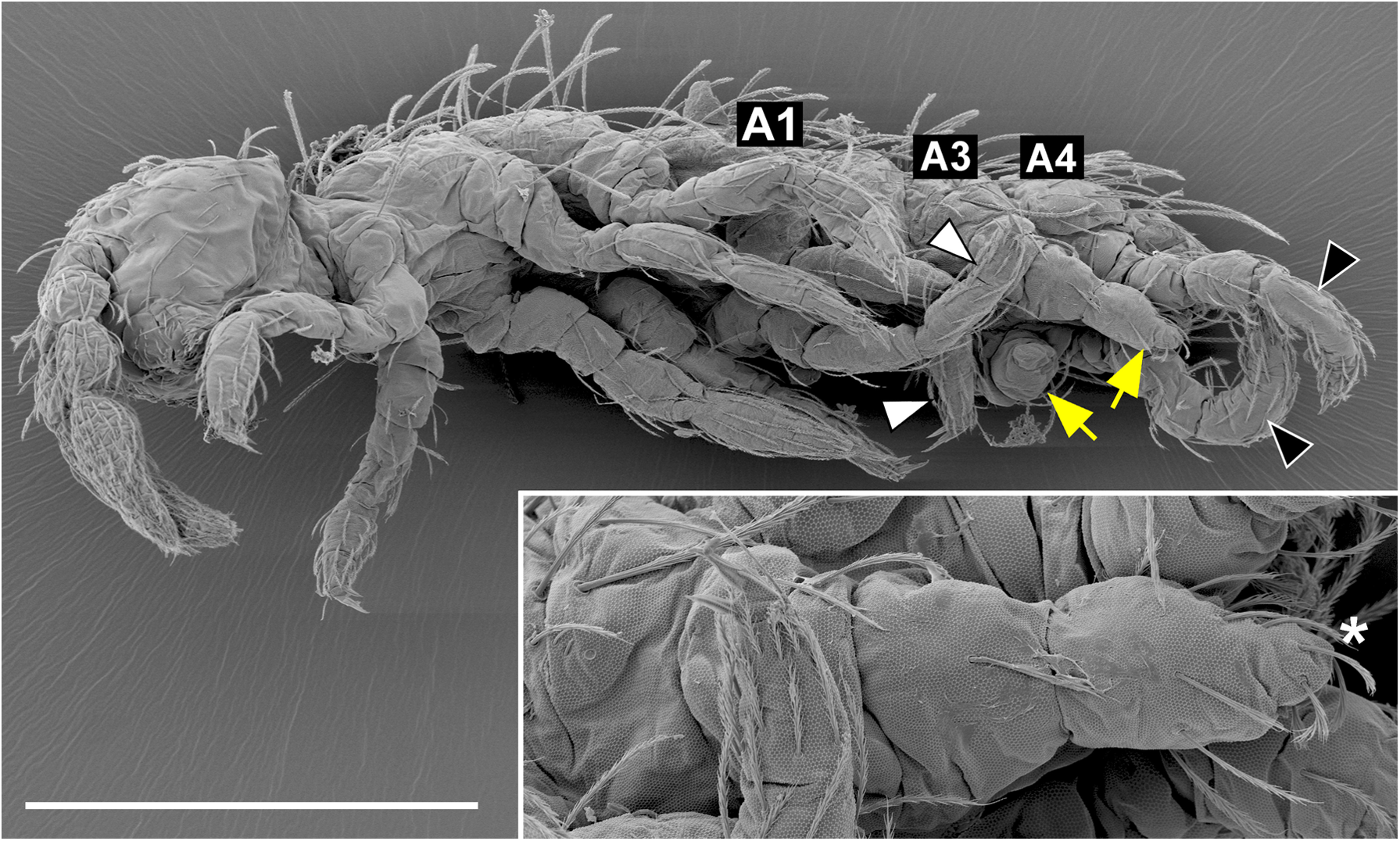

Supplement: Supplementary file 10 — Authors’ original file for figure 6 [file 13227_2013_140_MOESM10_ESM.tif]

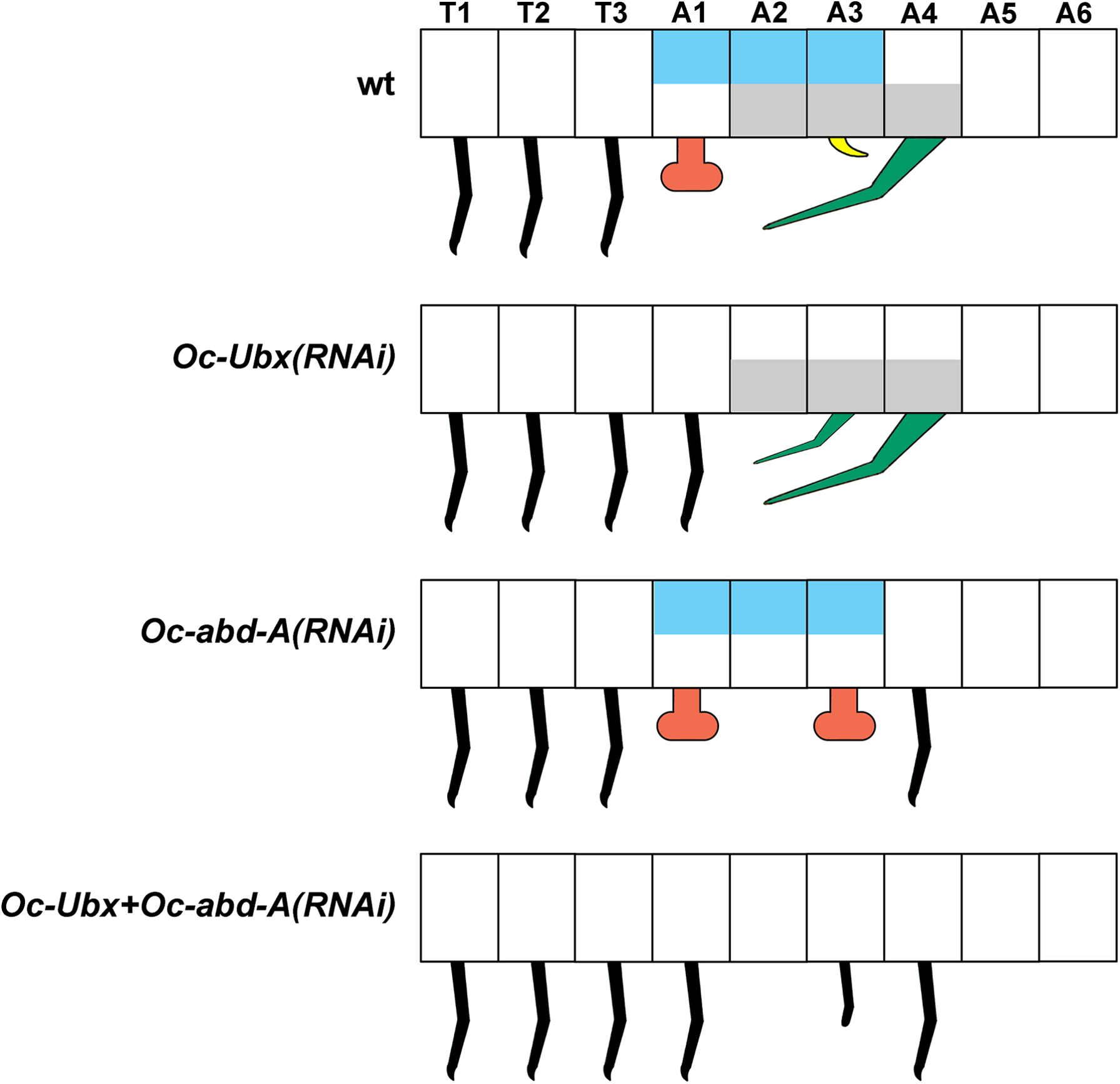

Supplement: Supplementary file 11 — Authors’ original file for figure 7 [file 13227_2013_140_MOESM11_ESM.tif]
